# Bioactive Compounds and Antioxidant Capacity of Wild Edible Asparagus in the Iğdır Plain

**DOI:** 10.1002/fsn3.70849

**Published:** 2025-08-28

**Authors:** Eren Özden, Adnan Aydin, Kaan Hürkan, Mehmet Nuri Atalar, Ayşe Türkhan

**Affiliations:** ^1^ Department of Horticulture, Faculty of Agriculture University of Iğdır Iğdır Türkiye; ^2^ Department of Horticulture and Agronomy, Faculty of Agriculture Kyrgyz‐Turkish Manas University, Chyngyz Aitmatov Campus (Djal) Bishkek Kyrgyz Republic; ^3^ Department of Agricultural Biotechnology, Faculty of Agriculture University of Igdir Iğdır Türkiye; ^4^ Department of Nutrition and Dietetics, Faculty of Health Sciences University of Iğdır Iğdır Türkiye; ^5^ Department of Chemistry and Chemical Processing Technologies, Technical Sciences Vocational School University of Igdir Iğdır Türkiye

**Keywords:** amino acids, antioxidant activity, *Asparagus* spp., nutritional composition, phenolic acids, wild edible plants

## Abstract

Worldwide, there is a growing interest in the consumption of wild edible plants that possess high nutritional value and health‐promoting bioactive compounds. Asparagus stands out among these plants due to its cultivated varieties as well as its wild forms. In Türkiye, asparagus consumption has increased more than tenfold over the past 5 years, and it is estimated that nearly half of the consumed asparagus comes from wild species obtained from local markets. Wild asparagus from the Iğdır Plain is in high demand locally and exported to nearby regions. This study was conducted to determine the biochemical composition and evaluate the antioxidant capacities of wild edible asparagus naturally distributed in the Iğdır Plain. A total of 35 wild asparagus spear samples were collected from different regions and analyzed for specific phenolic acids, flavonols, free amino acids, ascorbic acid, total saponin content, and antioxidant activities using DPPH and ABTS methods. According to the results, the wild asparagus samples collected from the Iğdır Plain exhibited higher levels of total phenolic acids, flavonols, amino acids, ascorbic acid, and saponins compared to the commercial cultivated variety. However, when evaluated in terms of antioxidant capacity, the wild samples showed lower activity than the commercial variety in both DPPH and ABTS assays. Statistical analyses revealed a significant level of variation among the wild asparagus genotypes in the region. Notably, samples collected from Iğdır city center, as well as regions A1, A15, A22, A24, and A27, were found to be particularly rich in nutritional and health‐related compounds. This study presents the first scientific data on the biochemical composition and antioxidant capacity of wild edible asparagus naturally growing in the Iğdır region. Furthermore, it is suggested that collecting seeds from promising regions and cultivating these genotypes could help prevent the extinction of these valuable wild species due to overharvesting.

## Graphical abstract

This study revealed significant biochemical diversity among wild asparagus populations in Iğdır, with generally higher phenolic acids, flavonoids (especially rutin and chlorogenic acid), antioxidant capacity, and saponin contents compared to the commercial cultivar. PCA and clustering analyses showed distinct groupings based on biochemical profiles, especially highlighting Iğdır and Tuzluca samples. The commercial variety often differed significantly, forming separate clusters in most analyses.

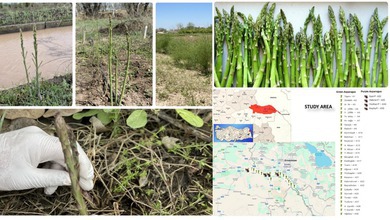

## 1 Introduction

The genus Asparagus belonging to the Asparagaceae family includes around 300 species worldwide, while in Türkiye, 11 species and 12 taxa (5 of which are endemic) naturally grow (Davis 1984) Asparagus species, which originated from the Mediterranean coasts, are distributed across Europe, Anatolia, Asia, and Africa (Norup et al. 2015). The genus includes both monoecious and dioecious species, and all species found in Türkiye are dioecious (2n = 20) (Castro et al. 2013). While the aerial parts of the plant grow and die within the same year, the underground root system remains alive for many years (Özden et al. 2023).

Asparagus is a temperate‐climate crop that can be successfully cultivated across a broad geographic range, extending from western Africa to northern Europe. It grows best in sandy‐loamy soils, while cultivation in heavy or poorly drained soils poses significant risks, including crown rot and increased plant mortality. Depending on the variety and maintenance conditions, harvesting can continue for 12–15 years after the establishment of a plantation. Asparagus is relatively drought‐tolerant (Sterrett et al. 1990) and considered among the vegetable species tolerant to salinity levels below 0.3% (Cao et al. 2014). Wild asparagus species are thought to be even more tolerant to drought and salinity compared to cultivated asparagus. Therefore, it can be economically grown in areas with low annual rainfall or relatively saline soils.

Various species of the Asparagus genus are used in nutrition, pharmaceuticals, and as ornamental plants. While the fresh spears of many species are consumed as food, the species *A. officinalis* , which has been cultivated specifically for this purpose for over 2000 years (Townsend and Guest 1985), is the most widely used and is generally grown in open fields. Asparagus is an herbaceous perennial vegetable harvested for its spears, which are rich in nutrients and medicinal properties (Tomassoli et al. 2012; Drost 2018). It is considered nutritionally valuable due to its content of vitamins, steroidal saponins, flavonoids, minerals, and amino acids (Dawid and Hofmann 2012). Depending on species, variety, and cultivation method, spears can be green, white, or purple, and they can be consumed fresh or processed as canned or frozen products (Pegiou et al. 2019).

Wild plants that grow naturally without human cultivation and are consumed by both humans and animals have attracted considerable research interest. Edible wild plants are particularly valued for their nutritional constituents, including antioxidants and phenolic compounds. In recent years, the increasing focus on natural nutrition has heightened interest in these plants as sources of bioactive compounds (Mohammed et al. 2021; Özden et al. 2023). Nonetheless, it remains crucial to understand the biochemical profiles of wild‐harvested species. While some asparagus species may pose health risks if consumed excessively due to compounds such as saponins, others may possess higher concentrations of beneficial substances compared to their cultivated counterparts. So, excessive intake of saponins can also cause damage to metabolic organs such as the liver and kidneys (Cao et al. 2024).

China, Peru, and Mexico are the leading asparagus‐producing countries worldwide. While Peru ranks as the largest exporter, the United States and Germany are the primary importers (FAO 2023). In Türkiye, approximately 2387 tons of asparagus ( *Asparagus officinalis* ) were produced in 2024 on an area of 314.5 ha (TUİK 2025). In comparison, this figure was only 174 tons in 2020. With the growing awareness of its health benefits and recognition as a high‐value food, both consumption and production are steadily increasing (Özden et al. 2023). In various regions of Türkiye, both *A. officinalis* and other non‐cultivated species naturally grow. Besides recorded production data, wild asparagus is also collected and consumed in different regions of Türkiye. For example, in Aegean and Mediterranean regions, wild asparagus collected from nature during March–May is not only enjoyed by locals but also sold at relatively high prices in local markets (Özden et al. 2023). A similar situation applies to the easternmost region of Türkiye, particularly the Iğdır Plain.

Ecologically, Iğdır differs from surrounding provinces and exhibits microclimatic characteristics. Instead of a harsh continental climate, it has a milder climate, which increases the number of species and varieties that can be cultivated or naturally grow. Species of the Asparagus genus (especially *Asparagus officinalis* ) have adapted to the Iğdır ecology and can be found in almost all settlements, especially along the Aras River. From early April to the end of July, wild asparagus is collected and consumed by the local population or sold in markets as a source of income. In addition to the domestic market, the region's asparagus is believed to have high antioxidant content and is in demand from nearby cities and even neighboring countries (Özden et al. 2023).

In this study, the phenolic compound contents, antioxidant capacities, amino acid profiles, and saponin levels of naturally growing wild asparagus in the Iğdır Plain were investigated. The aim of the study is to provide the first nutritional and health‐related data on these edible asparagus spears found in Iğdır Plain, which is the largest microclimatic region in Türkiye and ecologically isolated from neighboring provinces. By comparing the basic biochemical properties of the locally consumed and marketed wild asparagus with commercially available asparagus obtained from national markets and existing literature data, a comprehensive assessment of their status is intended.

## 2 Materials and Methods

### 2.1 Materials

**FIGURE 1:**
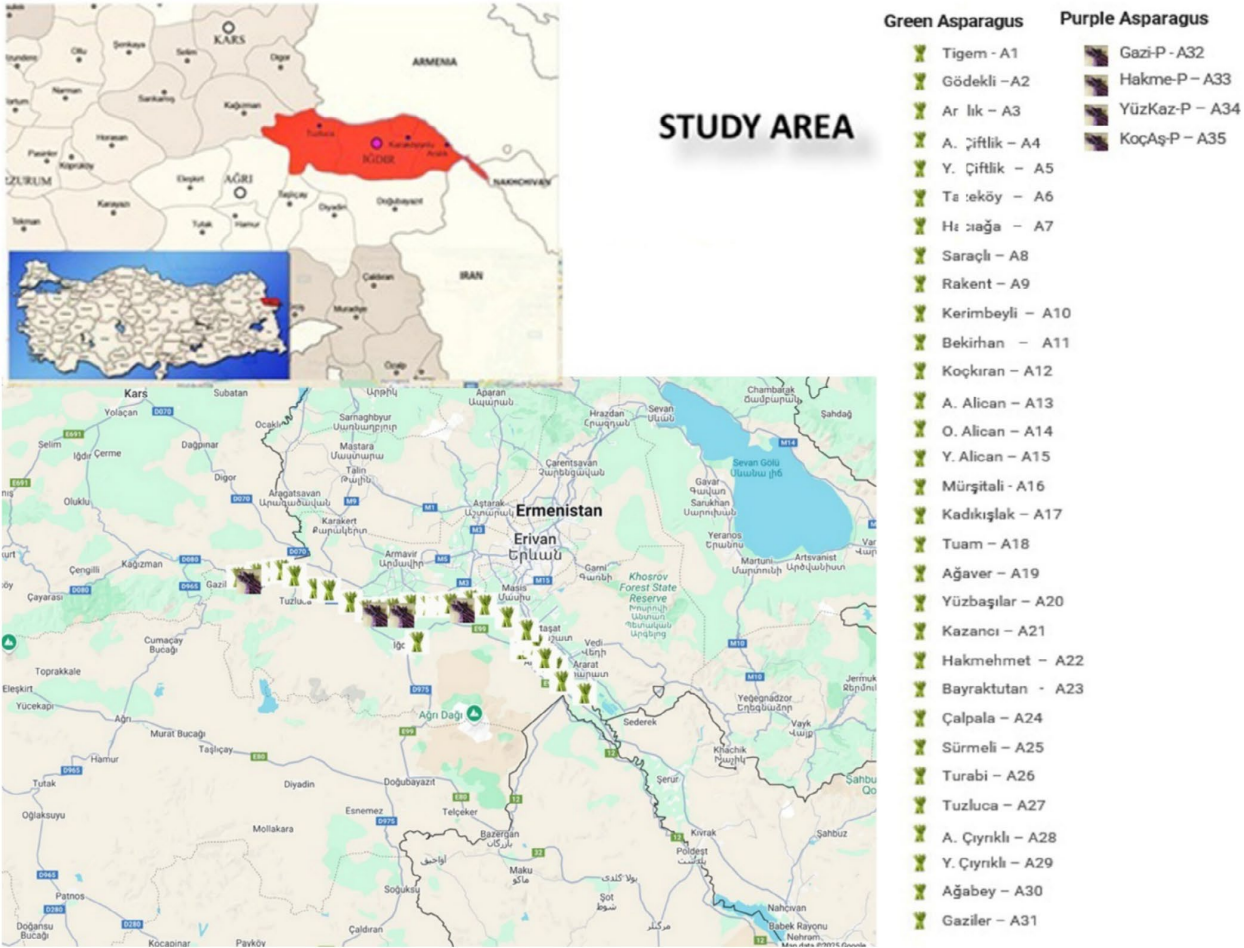
The regions along the Aras River in Iğdır Province where asparagus spears are collected.

Asparagus plants in the Iğdır Plain were initially marked in September 2021. To coincide with the period of peak spear emergence, samples were collected on March 20, 2022, between 05:00 and 18:00, from 35 different locations (31 of green, 4 of purple) across four districts (Iğdır city, Aralık, Karakoyunlu, and Tuzluca) (Table 1; Figure 1). From each site, edible‐sized spears (20–25 cm) were harvested in approximately 200 g quantities. Spears were collected from three different plants per location. To prevent moisture loss, the harvested samples were placed between moist paper towels and stored at −20°C on the same day until further analysis. For comparison, commercially available asparagus samples were also purchased from national supermarket chains. These samples were likewise stored at −20°C until use.

**TABLE 1:** Regions where asparagus spears are collected.

Aralık		Karakoyunlu		Iğdır		Tuzluca		Commercial cultivar
Region	Code	Region	Code	Region	Code	Region	Code	
Tigem	A1	Kerimbeyli	A10	Kadıkışlak	A17	Sürmeli	A25	A36
Gödekli	A2	Bekirhan	A11	Tuam	A18	Turabi	A26	
Aralık	A3	Koçkıran	A12	Ağaver	A19	Tuzluca	A27	
Aşağı Çiftlik	A4	Aşağı Alican	A13	Yüzbaşılar	A20	Aşağı çıyrıklı	A28	
Yukarı Çiftlik	A5	Orta Alican	A14	Kazancı	A21	Yukarı Çıyrıklı	A29	
Tazeköy	A6	Yukarı Alican	A15	Hakmehmet	A22	Ağabey	A30	
Hacıağa	A7	Mürşitali	A16	Bayraktutan	A23	Gaziler	A31	
Saraçlı	A8	KoçAş‐Purple	A35	Çalpala	A24	Gaziler‐purple	A32	
Ramazankent	A9			Hakme‐purple	A33			
				YüzKaz‐purple	A34			

*Note:* In the numbering of the regions; blue color: Aralık, red color: Karakoyunlu, purple color: Iğdır, yellow color: Tuzluca and green color: represents the commercial variety.

### 2.2 Methods

In this study, a single sample preparation procedure was applied to all asparagus spear samples, and the liquid extracts obtained were used for all subsequent analyses. From each location, 10 g of asparagus spears were weighed and ground in porcelain mortars with the addition of liquid nitrogen. The ground samples were then transferred into sterile 50 mL Falcon tubes, and 20 mL of methanol (LC–MS/MS grade) was added. To ensure thorough contact between the methanol and the plant material, the samples were incubated in the dark at +5°C for 24 h. After this period, the samples were homogenized at 600 rpm for 1 min using a homogenizer and incubated again at +5°C in the dark for 24 h. The next day, the samples were filtered through Whatman No. 3 filter paper. The filtrates were centrifuged at +4°C, 14,000 rpm for 30 min. After centrifugation, the supernatants were evaporated at +40°C for 15 min using a rotary evaporator to remove the methanol. The remaining residue was reconstituted with 20 mL of methanol and incubated again at +5°C in the dark for 24 h. On the following day, the samples were once more centrifuged at +4°C, 14,000 rpm for 30 min, and the supernatants were carefully pipetted. Finally, the samples were filtered through 0.45 μm SPE filters, making them ready for further biochemical analyses.

#### 2.2.1 Determination of Phenolic Acids and Flavanols

Phenolic acids and flavanols were analyzed both qualitatively and quantitatively using an Agilent LC–MS/MS system (Agilent 1290 Infinity LC–MS/MS, 6460 Triple Quadrupole LC/MS–MS) equipped with a Bin Pump Infinity DAD 1290 detector (*λ* = 260 and 310 nm). Phenolic acids were identified using standards dissolved in methanol, following the method described by Kobus et al. (2009). The composition of flavonols was determined with slight modifications according to the method described by Gramza‐Michałowska et al. (2016). Chromatograms of the phenolic compounds obtained from the LC–MS/MS readings, along with location‐specific chromatographic profiles, can be found in Figures S1 and S2.

#### 2.2.2 Determination of Total Antioxidant Capacity and Ascorbic Acid Content

The antioxidant capacity of asparagus spears was estimated using DPPH, ABTS, and ferric reducing power assays. The DPPH procedure, as described by Amarowicz et al. (2007), is based on the decrease in absorbance of a DPPH solution at *λ* = 517 nm in the presence of free radicals. DPPH radical scavenging activity was expressed as the percentage of radicals scavenged, EC_50_, and antiradical efficiency (AE), calculated as AE = 1/EC_50_.

The ABTS radical cation decolorization assay was performed according to Re et al. (1999) and was calculated based on spectrophotometric measurements at *λ* = 734 nm. The results were expressed in the same manner as DPPH: inhibition %, EC_50_, and antiradical efficiency (AE). The absorbance of the final mixture for the reducing power assay was measured at 700 nm. For calibration curves of the DPPH and ABTS assays, refer to Figures S3 and S4.

Ascorbic acid content was measured using 2,6‐dichlorophenolindophenol. The extraction of ascorbic acid was carried out by mixing 5 mL of 5% (w/v) trichloroacetic acid solution with 1 mL of green asparagus juice. After centrifugation, the collected supernatant was titrated with a 0.1% (w/v) aqueous solution of sodium 2,6‐dichlorophenolindophenol, as described by Chen et al. (2015).

#### 2.2.3 Determination of Free Amino Acids

The determination of the free amino acid composition of asparagus spears was based on HPLC methods described by Aristoy and Toldra (1991) with minor modifications. For the analysis, a single UV detector and a Zorbax Eclipse‐AAA 4.6 × 150 mm, 3.5 μm column (Agilent PN 963400–902) were used, operated on an Agilent 1290 Infinity LC–MS/MS (6460 Triple Quadrupole LC/MS–MS) system.

In the chromatographic system, the mobile phases were as follows:

Mobile Phase A: 40 mM NaH_2_PO_4_ (pH 7.8), and Mobile Phase B: A mixture of acetonitrile (ACN): methanol (MeOH): water in a ratio of 45:45:10 (v/v/v). The flow rate of the mobile phase was set at 2 mL/min, and the column temperature was maintained at 40°C. For chromatograms of the detected free amino acids and their region‐specific distributions, refer to Figures S5 and S6.

#### 2.2.4 Determination of Total Saponins

The diosgenin content in asparagus spears was determined using an LC–MS/MS system to compare it with the total saponin content. The total saponin content was spectrophotometrically quantified with minor modifications based on the method described by Medina‐Meza et al. (2016). To 1 mL of reagent mixture (ice‐cold acetic acid/sulfuric acid, 1:1 v/v), 0.25 mL of the saponin extract from each sample was added. The mixture was vortexed and incubated in a water bath at 60°C for 30 min, followed by cooling. Diosgenin (0–1.000 μg/mL) was used as the standard. The total saponin content was expressed as mg g^−1^ diosgenin equivalent (DE). For the standard chromatogram of diosgenin in the LC–MS/MS system and the steroidal diosgenin calibration curve used in the calculation of saponins, refer to Figure S7.

### 2.3 Statistical Analysis

All measurements were performed in triplicate, and the results were expressed as mean ± standard error of mean (SEM) (IBM Corp., Armonk, NY, USA). In this study, statistical analyses were conducted using Duncan's multiple range test to determine significant differences between locations at *p* < 0.05. Cluster constellation plot and heatmap analysis were performed using JMP 14.3 statistical software. Furthermore, principal component analysis and radar map were performed with OriginPro19 software.

## 3 Results

### 3.1 Phenolic Acid Contents in Asparagus Spears

Based on phenolic acid analyses performed on asparagus spears using the LC–MS/MS system, 4‐hydroxybenzoic acid and vanillic acid were not detected. While shikimic acid, gallic acid, protocatechuic acid, caffeic acid, and sinapic acid could be quantified in spears collected from certain regions, they could not be quantified in samples from other regions. The amounts of chlorogenic acid, p‐coumaric acid, and trans‐ferulic acid were determined in asparagus samples collected from all regions (Table 2).

**TABLE 2:** Phenolic acid contents in asparagus spears (mg/100 g).

Region	Shikimic acid	Gallic acid	Protocatechuic acid	Chlorogenic acid	Caffeic acid	*p*‐coumaric acid	Trans‐ferulic acid	Sinapic acid
A1	15.661 ± 1.6	ND	ND	141.70 ± 2.1	ND	0.603 ± 0.007	8.507 ± 0.18	0.158 ± 0.011
A2	ND	ND	ND	238.51 ± 3.6	ND	0.432 ± 0.005	**5.955 ± 0.13**	ND
A3	15.234 ± 1.5	ND	ND	336.78 ± 5.1	ND	0.837 ± 0.010	11.187 ± 0.24	ND
A4	17.436 ± 1.7	ND	1.401 ± 0.09	106.53 ± 1.6	ND	**0.349 ± 0.004**	6.703 ± 0.14	ND
A5	16.571 ± 1.6	ND	**0.326 ± 0.02**	**76.02 ± 1.1**	ND	0.726 ± 0.009	9.638 ± 0.21	ND
A6	16.696 ± 1.7	ND	3.959 ± 0.26	718.93 ± 10.8	2.762 ± 0.066	3.931 ± 0.047	41.728 ± 0.89	ND
A7	19.432 ± 1.9	ND	1.541 ± 0.10	359.88 ± 5.4	ND	1.064 ± 0.013	8.345 ± 0.18	ND
A8	16.669 ± 1.7	ND	2.564 ± 0.17	368.65 ± 5.5	**0.261 ± 0.006**	0.680 ± 0.008	13.476 ± 0.29	2.709 ± 0.192
A9	ND	**0.005 ± 0.00**	1.686 ± 0.11	100.63 ± 1.5	ND	1.064 ± 0.013	22.356 ± 0.48	ND
A10	14.550 ± 1.4	ND	1.370 ± 0.09	109.24 ± 1.6	ND	1.565 ± 0.019	32.898 ± 0.70	ND
A11	13.750 ± 1.4	ND	2.269 ± 0.15	155.66 ± 2.3	ND	0.541 ± 0.006	14.680 ± 0.31	2.333 ± 0.166
A12	17.279 ± 1.7	0.006 ± 0.00	2.969 ± 0.20	621.31 ± 9.3	2.514 ± 0.060	1.382 ± 0.017	18.548 ± 0.40	ND
A13	28.022 ± 2.8	ND	3.739 ± 0.25	220.75 ± 3.3	1.351 ± 0.032	1.303 ± 0.016	19.110 ± 0.41	**0.031 ± 0.002**
A14	22.963 ± 2.3	ND	2.442 ± 0.16	764.60 ± 11.5	9.380 ± 0.225	1.526 ± 0.018	8.893 ± 0.19	ND
A15	14.411 ± 1.4	0.010 ± 0.00	4.446 ± 0.29	1968.9 ± 29.5	7.610 ± 0.183	1.971 ± 0.024	35.025 ± 0.75	1.266 ± 0.090
A16	15.785 ± 1.6	ND	3.439 ± 0.23	1319.5 ± 19.8	8.633 ± 0.207	3.354 ± 0.040	35.429 ± 0.76	1.082 ± 0.077
A17	23.235 ± 2.3	0.018 ± 0.00	3.397 ± 0.22	978.83 ± 14.7	5.192 ± 0.125	2.066 ± 0.025	23.889 ± 0.51	2.277 ± 0.162
A18	16.197 ± 1.6	0.015 ± 0.00	3.306 ± 0.22	**2131.2 ± 32.0**	16.691 ± 0.401	1.986 ± 0.024	**60.174 ± 1.29**	11.261 ± 0.800
A19	21.174 ± 2.1	ND	3.254 ± 0.21	1555.9 ± 23.3	6.127 ± 0.147	1.255 ± 0.015	17.280 ± 0.37	1.657 ± 0.118
A20	15.232 ± 1.5	0.053 ± 0.00	1.523 ± 0.10	670.82 ± 10.1	0.772 ± 0.019	1.392 ± 0.017	35.612 ± 0.76	ND
A21	13.888 ± 1.4	**0.029 ± 0.00**	4.464 ± 0.29	602.47 ± 9.0	1.516 ± 0.036	1.504 ± 0.018	22.696 ± 0.49	ND
A22	13.531 ± 1.3	ND	2.214 ± 0.15	1838.9 ± 27.6	**21.717 ± 0.0521**	3.975 ± 0.048	31.005 ± 0.66	1.711 ± 0.121
A23	14.926 ± 1.5	ND	2.373 ± 0.16	555.27 ± 8.3	1.188 ± 0.029	1.596 ± 0.019	16.533 ± 0.35	ND
A24	**28.695 ± 2.8**	ND	4.618 ± 0.30	822.05 ± 12.3	3.385 ± 0.081	**6.239 ± 0.075**	48.906 ± 1.05	ND
A25	14.893 ± 1.5	ND	2.353 ± 0.16	1101.8 ± 16.5	6.424 ± 0.154	2.788 ± 0.033	18.205 ± 0.39	2.523 ± 0.179
A26	14.994 ± 1.5	ND	3.450 ± 0.23	1228.9 ± 18.4	5.910 ± 0.142	3.166 ± 0.038	32.741 ± 0.70	**12.337 ± 0.876**
A27	15.143 ± 1.5	ND	**8.226 ± 0.54**	58.35 ± 0.9	0.934 ± 0.022	1.114 ± 0.013	16.396 ± 0.35	3.294 ± 0.234
A28	13.769 ± 1.4	ND	4.134 ± 0.27	892.26 ± 13.4	0.917 ± 0.022	2.407 ± 0.029	40.276 ± 0.86	2.647 ± 0.188
A29	17.210 ± 1.7	0.024 ± 0.00	6.119 ± 0.40	419.06 ± 6.3	0.962 ± 0.023	1.332 ± 0.016	24.989 ± 0.53	0.552 ± 0.039
A30	**13.375 ± 1.3**	ND	7.012 ± 0.46	465.08 ± 7.0	2.943 ± 0.071	2.747 ± 0.033	30.619 ± 0.66	1.420 ± 0.101
A31	14.282 ± 1.4	ND	2.401 ± 0.16	506.57 ± 7.6	5.593 ± 0.134	0.752 ± 0.009	14.747 ± 0.32	ND
A32	17.888 ± 1.8	ND	7.431 ± 0.49	111.48 ± 1.7	1.425 ± 0.034	2.166 ± 0.026	22.622 ± 0.48	3.155 ± 0.224
A33	14.918 ± 1.5	ND	4.370 ± 0.29	1004.7 ± 15.1	6.857 ± 0.165	2.046 ± 0.025	10.965 ± 0.23	1.544 ± 0.110
A34	22.579 ± 2.2	ND	3.815 ± 0.25	899.89 ± 13.5	3.141 ± 0.075	1.721 ± 0.021	32.364 ± 0.69	4.066 ± 0.289
A35	19.511 ± 1.9	ND	3.460 ± 0.23	533.23 ± 8.0	2.286 ± 0.055	1.813 ± 0.022	19.598 ± 0.42	1.318 ± 0.094
A36	13.621 ± 1.3	ND	1.473 ± 0.10	246.86 ± 3.7	6.587 ± 0.158	5.476 ± 0.066	40.083 ± 0.86	2.552 ± 0.181

*Note:* ±: SEM, Dark colors indicate the highest value, light colors indicate the lowest value. In the numbering of the regions; blue color: Aralık, red color: Karakoyunlu, purple color: Iğdır, yellow color: Tuzluca and green color: represents the commercial variety.

Abbreviation: ND, Not determined.

The highest shikimic acid content was determined in region A24 with 28.695 mg/100 g, while the lowest was found in region A30 with 13.375 mg/100 g. Considering the cities from which the spears were collected, shikimic acid levels ranged between 18.438 and 15.194 mg/100 g, with the highest average observed in the city center of Iğdır. In general, the average shikimic acid content of the cities was higher than that of the commercial sample A36, which had a content of 13.621 mg/100 g (Table 2).

Among the regions where gallic acid was detected, the highest content was found in region A20, and the lowest in region A9. Based on cities, the highest average gallic acid content was determined in spears collected from Iğdır. Gallic acid was not detected in samples collected from the city of Aralık or in the commercial cultivar (Table 2).

Protocatechuic acid content varied between 8.226 and 0.326 mg/100 g across the regions. The highest content was determined in region A27, while the lowest was found in region A5. On a city basis, the highest average protocatechuic acid content was observed in Tuzluca, whereas the lowest was in the commercial variety (Table 2).

Although there were large differences in chlorogenic acid content across regions, the highest value was calculated as 2131.22 mg/100 g in region A18, and the lowest as 76.02 mg/100 g in region A5. At the city level, the highest average chlorogenic acid content was found in samples from Iğdır, while the lowest was observed in samples from the commercial cultivar (Table 2).

When examining the contents of caffeic acid, another compound analyzed, the levels were found to range between 0.261 and 21.717 mg/100 g across the regions. The highest caffeic acid content was calculated in region A22, while the lowest was in region A8. On a city basis, the highest average caffeic acid content was observed in samples from Iğdır, and the lowest in samples from Aralık (Table 2).

The highest *p*‐coumaric acid content in asparagus spears was found in region A24, while the lowest was observed in region A4. The highest average *p*‐coumaric acid content was calculated in the commercial cultivar, whereas the lowest was observed in samples collected from Aralık (Table 2).

Regarding trans‐ferulic acid contents, the highest value was observed in region A18 with 60.174 mg/100 g, and the lowest in region A2 with 5.955 mg/100 g. The highest average trans‐ferulic acid content was found in the commercial cultivar, while the lowest was observed in samples from Aralık (Table 2).

According to regions, the highest sinapic acid content was determined in region A26 with 12.337 mg/100 g, and the lowest in region A13 with 0.031 mg/100 g. On a city basis, the highest average sinapic acid content was observed in Iğdır, while the lowest was found in Karakoyunlu (Table 2).

### 3.2 Flavanol Contents in Asparagus Spears

Based on flavanol analyses performed on asparagus spears using the LC–MS/MS system, kaempferol was not detected, while fisetin was identified only in samples from region A15, with a concentration of 3.839 mg/100 g. Quercimeritrin, scutellarin, astragalin, quercetin, and naringenin could be quantified in spear samples from some regions, whereas they could not be quantified in samples from other regions. The amounts of coumarin, hyperoside, rutin, isoquercetin, chrysin, and flavone were determined in asparagus samples collected from all regions (Table 3).

**TABLE 3:** Flavanol contents in asparagus spears (mg/100 g).

Region	Kuersime ritrin	Coumarin	Scutellarin	Hyperocyte	Rutin	Isoquercetin	Astragalin	Quercetin	Naringenin	Krisin	Flavon
A1	2.27 ± 0.027	**22.55 ± 0.34**	ND	41.67 ± 3.4	424.96 ± 4.7	14.57 ± 0.17	0.079 ± 0.0017	ND	ND	**140.51 ± 8.3**	**1.881 ± 0.0169**
A2	ND	9.53 ± 0.14	ND	35.24 ± 2.9	572.57 ± 6.3	12.33 ± 0.15	0.016 ± 0.0004	ND	0.390 ± 0.021	75.03 ± 4.4	0.662 ± 0.0060
A3	ND	15.42 ± 0.23	0.17 ± 0.002	20.32 ± 1.7	349.87 ± 3.8	7.12 ± 0.09	ND	ND	0.732 ± 0.040	**70.09 ± 4.1**	0.635 ± 0.0057
A4	ND	0.81 ± 0.01	**0.16 ± 0.002**	**9.01 ± 0.7**	121.26 ± 1.3	**3.17 ± 0.04**	**0.002 ± 0.0001**	ND	0.065 ± 0.004	79.36 ± 4.7	**0.527 ± 0.0047**
A5	ND	15.22 ± 0.23	ND	26.24 ± 2.2	413.15 ± 4.5	9.18 ± 0.11	0.039 ± 0.0009	**0.10 ± 0.002**	**0.063 ± 0.003**	80.40 ± 4.7	0.798 ± 0.0072
A6	**1.30 ± 0.016**	2.23 ± 0.03	ND	79.00 ± 6.5	2006.16 ± 22.1	27.60 ± 0.33	0.109 ± 0.0024	1.92 ± 0.035	1.340 ± 0.074	71.78 ± 4.2	0.560 ± 0.0050
A7	ND	0.99 ± 0.01	ND	23.16 ± 1.9	350.20 ± 3.9	8.11 ± 0.10	0.071 ± 0.0016	ND	0.932 ± 0.051	76.40 ± 4.5	0.677 ± 0.0061
A8	ND	0.83 ± 0.01	1.02 ± 0.014	16.41 ± 1.3	218.99 ± 2.4	5.76 ± 0.07	ND	2.87 ± 0.052	1.172 ± 0.064	95.78 ± 5.7	0.740 ± 0.0067
A9	ND	**0.79 ± 0.01**	1.39 ± 0.019	22.08 ± 1.8	315.31 ± 3.5	7.74 ± 0.09	0.041 ± 0.0009	ND	0.720 ± 0.040	82.46 ± 4.9	0.727 ± 0.0065
A10	ND	0.85 ± 0.01	1.80 ± 0.025	40.36 ± 3.3	601.89 ± 6.6	14.11 ± 0.17	0.068 ± 0.0015	ND	**1.577 ± 0.087**	74.03 ± 4.4	0.693 ± 0.0062
A11	ND	0.81 ± 0.01	0.31 ± 0.004	23.82 ± 2.0	240.68 ± 2.6	8.34 ± 0.10	0.024 ± 0.0005	ND	1.000 ± 0.055	87.41 ± 5.2	0.682 ± 0.0061
A12	ND	2.27 ± 0.03	1.71 ± 0.024	50.79 ± 4.2	689.24 ± 7.6	17.75 ± 0.21	0.013 ± 0.0003	ND	0.845 ± 0.046	99.39 ± 5.9	0.773 ± 0.0070
A13	1.83 ± 0.022	1.00 ± 0.02	1.05 ± 0.015	16.01 ± 1.3	148.26 ± 1.6	5.62 ± 0.07	0.049 ± 0.0011	2.40 ± 0.043	0.065 ± 0.004	90.39 ± 5.3	0.933 ± 0.0084
A14	ND	1.31 ± 0.02	2.90 ± 0.041	35.79 ± 2.9	375.70 ± 4.1	12.52 ± 0.15	0.061 ± 0.0013	1.32 ± 0.024	0.459 ± 0.025	89.48 ± 5.3	0.863 ± 0.0078
A15	ND	1.49 ± 0.02	3.02 ± 0.042	**164.94 ± 13.5**	**2456.06 ± 27.0**	**57.59 ± 0.69**	1.253 ± 0.0276	8.98 ± 0.162	1.194 ± 0.066	71.55 ± 4.2	0.738 ± 0.0066
A16	ND	1.58 ± 0.02	1.75 ± 0.025	85.94 ± 7.0	910.16 ± 10.0	30.02 ± 0.36	**2.025 ± 0.0446**	3.35 ± 0.060	0.267 ± 0.015	84.78 ± 5.0	0.634 ± 0.0057
A17	ND	1.33 ± 0.02	1.58 ± 0.022	47.30 ± 3.9	621.47 ± 6.8	16.54 ± 0.20	0.193 ± 0.0042	0.99 ± 0.018	0.071 ± 0.004	86.15 ± 5.1	0.667 ± 0.0060
A18	2.18 ± 0.026	11.98 ± 0.18	ND	123.62 ± 10.1	2000.03 ± 22.0	43.17 ± 0.52	0.153 ± 0.0034	**9.90 ± 0.178**	0.260 ± 0.014	90.47 ± 5.3	0.996 ± 0.0090
A19	ND	12.96 ± 0.19	1.29 ± 0.018	96.67 ± 7.9	1321.80 ± 14.5	33.77 ± 0.41	0.150 ± 0.0033	3.12 ± 0.056	0.332 ± 0.018	94.06 ± 5.5	0.644 ± 0.0058
A20	ND	2.70 ± 0.04	2.53 ± 0.035	43.40 ± 3.6	633.96 ± 7.0	15.18 ± 0.18	0.160 ± 0.0035	1.26 ± 0.023	0.296 ± 0.016	86.99 ± 5.1	0.641 ± 0.0058
A21	ND	1.81 ± 0.03	1.74 ± 0.024	81.42 ± 6.7	1479.04 ± 16.3	28.44 ± 0.34	0.216 ± 0.0048	0.25 ± 0.004	0.256 ± 0.014	87.66 ± 5.2	0.678 ± 0.0061
A22	ND	2.23 ± 0.03	2.60 ± 0.036	93.23 ± 7.6	1579.23 ± 17.4	32.56 ± 0.39	0.361 ± 0.0079	6.73 ± 0.121	0.892 ± 0.049	90.64 ± 5.3	0.667 ± 0.0060
A23	ND	1.14 ± 0.02	3.76 ± 0.053	31.62 ± 2.6	457.10 ± 5.0	11.06 ± 0.13	0.131 ± 0.0029	1.17 ± 0.021	ND	95.80 ± 5.7	0.764 ± 0.0069
A24	ND	2.91 ± 0.04	**4.01 ± 0.056**	110.68 ± 9.1	1904.90 ± 21.0	38.65 ± 0.46	0.334 ± 0.0073	1.06 ± 0.019	0.828 ± 0.046	77.26 ± 4.6	0.728 ± 0.0066
A25	ND	1.64 ± 0.02	2.18 ± 0.030	123.36 ± 10.1	2076.57 ± 22.8	43.08 ± 0.52	0.123 ± 0.0027	1.60 ± 0.029	0.724 ± 0.040	82.30 ± 4.9	0.956 ± 0.0086
A26	ND	1.01 ± 0.02	2.77 ± 0.039	94.85 ± 7.8	1521.78 ± 16.7	33.13 ± 0.40	0.057 ± 0.0013	6.30 ± 0.113	0.610 ± 0.034	88.70 ± 5.2	0.551 ± 0.0050
A27	ND	0.85 ± 0.01	1.52 ± 0.021	14.24 ± 1.2	**117.45 ± 1.3**	4.99 ± 0.06	0.006 ± 0.0001	ND	0.423 ± 0.023	81.12 ± 4.8	0.803 ± 0.0072
A28	2.11 ± 0.025	0.90 ± 0.01	1.24 ± 0.017	15.18 ± 1.2	155.11 ± 1.7	5.33 ± 0.06	0.029 ± 0.0006	0.39 ± 0.007	0.734 ± 0.040	83.16 ± 4.9	0.658 ± 0.0059
A29	ND	1.08 ± 0.02	0.97 ± 0.014	42.64 ± 3.5	540.92 ± 6.0	14.91 ± 0.18	0.182 ± 0.0040	0.28 ± 0.005	0.476 ± 0.026	79.88 ± 4.7	0.801 ± 0.0072
A30	ND	1.00 ± 0.02	0.99 ± 0.014	22.27 ± 1.8	208.65 ± 2.3	7.80 ± 0.09	0.022 ± 0.0005	0.78 ± 0.014	0.335 ± 0.018	88.33 ± 5.2	0.812 ± 0.0073
A31	ND	0.87 ± 0.01	1.91 ± 0.027	59.24 ± 4.9	803.67 ± 8.8	20.70 ± 0.25	0.032 ± 0.0007	1.89 ± 0.034	0.928 ± 0.051	92.67 ± 5.5	0.863 ± 0.0078
A32	ND	0.86 ± 0.01	2.51 ± 0.035	23.46 ± 1.9	248.91 ± 2.7	8.22 ± 0.10	0.006 ± 0.0001	ND	0.866 ± 0.048	83.51 ± 4.9	0.80 ± 0.0072
A33	ND	1.72 ± 0.03	1.29 ± 0.018	58.53 ± 4.8	792.99 ± 8.7	20.45 ± 0.25	0.091 ± 0.0020	1.15 ± 0.021	0.263 ± 0.014	82.95 ± 4.9	0.830 ± 0.0075
A34	ND	1.31 ± 0.02	2.93 ± 0.041	54.91 ± 4.5	624.95 ± 6.9	19.19 ± 0.23	0.451 ± 0.0099	7.56 ± 0.136	1.519 ± 0.084	96.91 ± 5.7	0.620 ± 0.0056
A35	ND	1.07 ± 0.02	0.79 ± 0.011	41.57 ± 3.4	512.39 ± 5.6	14.54 ± 0.17	0.116 ± 0.0026	1.59 ± 0.029	ND	95.12 ± 5.6	0.710 ± 0.0064
A36	**7.68 ± 0.092**	1.61 ± 0.02	ND	30.37 ± 2.5	315.89 ± 3.5	10.63 ± 0.13	0.013 ± 0.0003	6.09 ± 0.110	0.550 ± 0.030	92.33 ± 5.4	1.090 ± 0.0098

*Note:* ±: SEM, Dark colors indicate the highest value, light colors indicate the lowest value. In the numbering of the regions; blue color: Aralık, red color: Karakoyunlu, purple color: Iğdır, yellow color: Tuzluca and green color: represents the commercial variety.

Abbreviation: ND. Not determined.

Among the regions where values could be obtained, quercimeritrin contents ranged between 7.68 and 1.30 mg/100 g. The highest quercimeritrin content was found in the commercial cultivar, while the lowest was determined in samples from region A6 (Table 3).

Coumarin contents were found to be highest in region A1 with 22.55 and lowest in region A9 with 0.79 mg/100 g. Considering city averages, the highest average coumarin content was observed in Aralık, while the lowest was recorded in Karakoyunlu (Table 3).

Scutellarin contents ranged from 4.01 mg/100 g in region K24 (the highest) to 0.16 mg/100 g in region A4 (the lowest). Based on city averages, the highest scutellarin content was found in Iğdır, while the lowest was observed in Aralık. Scutellarin was not detected in the commercial cultivar (Table 3).

Hyperoside content varied across regions, with the highest found in region A15 at 164.94 and the lowest in region A4 at 9.01 mg/100 g. Among city averages, the highest hyperoside content was observed in Iğdır, and the lowest in Aralık (Table 3).

Rutin contents ranged between 2456.06 and 117.45 mg/100 g across regions. The highest rutin content was found in region A15, while the lowest was in region A27. Based on city averages, the highest rutin content was observed in Iğdır, and the lowest in the commercial cultivar (Table 3).

Isoquercetin content was highest in region A15 at 57.59 and lowest in region A4 at 3.17 mg/100 g. The highest average isoquercetin content was found in Iğdır, and the lowest in Aralık (Table 3).

Astragalin contents varied between 2.025 and 0.002 mg/100 g across regions. The highest was in region A16 and the lowest in region A4. Based on cities, the highest average astragalin content was in Karakoyunlu, while the lowest was in the commercial cultivar.

Quercetin content ranged from 9.90 mg/100 g in region A18 to 0.10 mg/100 g in samples from region A5. The highest average quercetin content was found in the commercial cultivar, while the lowest average was observed in Aralık (Table 3).

Naringenin content was highest in region A10 with 1.577 and lowest in region A5 with 0.063 mg/100 g. By city averages, the highest naringenin content was observed in Karakoyunlu, and the lowest in Iğdır (Table 3).

Chrysin contents were found to be highest in region A1 with 140.51 and lowest in region A3 with 70.09 mg/100 g. The highest average chrysin content was detected in the commercial cultivar, while the lowest was in Tuzluca (Table 3).

Flavone content varied by region, with the highest in region A1 at 1.881 and the lowest in region A4 at 0.527 mg/100 g. The highest average flavone content was found in the commercial cultivar, while the lowest was recorded in Iğdır (Table 3).

### 3.3 Total Antioxidant and Ascorbic Acid Activities

In both DPPH and ABTS assays, the key values were calculated as IC50 (the antioxidant concentration required to reduce the initial DPPH or ABTS concentration by 50%) based on the generated graphs. The IC50 DPPH values measured in asparagus spears varied by region, ranging between 2.74 and 25.85 μg μL^−1^. Statistically, the highest IC50 value was found in samples from region A27, while the lowest was detected in samples from region A15. Based on city averages, the highest IC50 values were observed in samples collected from Aralık, while the lowest were found in samples from Iğdır (Table 4).

**TABLE 4:** DPPH, ABTS, and ascorbic acid values in asparagus spears by region.

Region	DPPH			ABTS			Ascorbic acid (μL L^−1^)
	IC50 (μg μL^−1^)	AR	% scavenging	IC50 (μg μL^−1^)	AR	% scavenging	
A1	**14.16 ± 0.36** h	**0.071 k–n**	**84.94 ± 2.15 h**	11.22 ± 0.16 i	**0.098 g**	69.31 ± 0.94 i	121.52 ± 2.51 q–s
A2	17.47 ± 0.31 d	0.057 n–q	104.84 ± 1.87 d	16.06 ± 0.28 c	0.062 no	96.34 ± 1.71 c	134.42 ± 0.98 lm
A3	16.53 ± 0.31 e	0.061 m–p	99.16 ± 1.85 e	15.79 ± 0.19 c	0.063 n	94.73 ± 1.13 c	163.18 ± 0.73 e
A4	22.58 ± 0.43 b	0.044 qr	135.48 ± 2.59 b	16.07 ± 0.15 c	0.062 no	96.39 ± 0.89 c	142.57 ± 1.56 hi
A5	11.42 ± 0.56 k	0.088 hi	68.54 ± 3.33 k	14.62 ± 0.39 d	0.068 mn	87.73 ± 2.37 d	179.62 ± 1.24 c
A6	6.97 ± 0.21 o	0.143 d	41.84 ± 1.26 o	7.86 ± 0.13 m	0.127 d	47.13 ± 0.81 m	117.53 ± 2.22 st
A7	11.19 ± 0.15 kl	0.089 g–i	67.14 ± 0.88 kl	12.31 ± 0.30 hi	0.081 h–j	73.88 ± 1.82 hi	109.11 ± 1.16 vw
A8	15.72 ± 0.21 fg	0.064 k–o	94.33 ± 1.24 fg	15.87 ± 0.15 c	0.063 n	95.23 ± 0.91 c	132.60 ± 3.04 mn
A9	16.29 ± 0.20 ef	0.061 m–p	97.72 ± 1.19 ef	15.63 ± 0.25 c	0.064 n	93.76 ± 1.51 c	135.54 ± 2.11 lm
A10	11.71 ± 0.16 k	0.085 h–j	70.24 ± 0.97 k	12.92 ± 0.16 gh	0.077 i–k	77.50 ± 0.98 gh	119.5 ± 0.49 r–t
A11	20.78 ± 0.38 c	0.048 p–r	124.65 ± 2.26 c	**20.88 ± 0.24 a**	**0.048 q**	**125.29 ± 1.45 a**	126.36 ± 1.86 op
A12	7.08 ± 0.18 o	0.141 d	42.46 ± 1.08 o	11.64 ± 0.15 i	0.086 h	69.86 ± 0.93 i	140.20 ± 1.69 i–k
A13	15.31 ± 0.22 g	0.065 k–n	91.83 ± 1.34 g	18.21 ± 0.23 b	0.055 p	109.27 ± 1.40 b	137.57 ± 0.69 j–l
A14	9.82 ± 0.26 m	0.102 fg	58.90 ± 1.55 m	14.16 ± 0.20 de	0.071 lm	84.94 ± 1.18 de	142.80 ± 0.37 hi
A15	**2.74 ± 0.12 *r***	**0.366 a**	**16.41 ± 0.71 *r***	**5.15 ± 0.08 *n***	**0.194 a**	**30.90 ± 0.48 *n***	125.71 ± 1.82 o–q
A16	6.93 ± 0.15 o	0.144 d	41.57 ± 0.89 o	7.74 ± 0.18 m	0.129 d	46.45 ± 1.07 m	113.02 ± 0.81 uv
A17	8.38 ± 0.26 *n*	0.119 e	50.25 ± 1.56 *n*	10.05 ± 0.01 j	0.100 g	60.29 ± 0.07 j	117.50 ± 0.57 st
A18	3.51 ± 0.21 q	0.285 b	21.03 ± 1.23 q	**5.45 ± 0.18 *n***	0.184 b	**32.67 ± 1.09 *n***	121.36 ± 0.56 q–s
A19	5.13 ± 0.14 *p*	0.195 c	30.80 ± 0.82 *p*	8.82 ± 0.11 l	0.113 e	52.90 ± 0.68 l	132.41 ± 0.95 mn
A20	8.42 ± 0.27 *n*	0.119 e	50.51 ± 1.62 *n*	12.31 ± 0.31 hi	0.081 h–j	73.85 ± 1.88 hi	145.13 ± 1.12 gh
A21	6.71 ± 0.35 o	0.149 d	40.26 ± 2.10 o	9.24 ± 0.29 kl	0.108 ef	55.44 ± 1.75 kl	159.63 ± 1.36 e
A22	7.15 ± 0.24 o	0.140 d	42.91 ± 1.47 o	7.68 ± 0.17 m	0.130d	46.05 ± 1.00 m	171.51 ± 0.98 d
A23	17.62 ± 0.20 d	0.057 n–q	105.74 ± 1.23 d	17.80 ± 0.18 b	0.056 op	106.79 ± 1.10 b	144.92 ± 0.67 gh
A24	6.90 ± 0.04 o	0.145 d	41.40 ± 0.27 o	7.22 ± 0.06 m	0.139 c	43.31 ± 0.37 v	133.74 ± 1.15 lm
A25	7.92 ± 0.22 *n*	0.126 e	47.50 ± 1.34 *n*	7.66 ± 0.35 m	0.131 d	45.97 ± 2.11 m	161.56 ± 1.30 e
A26	10.14 ± 0.07 m	0.099 gh	60.84 ± 0.44 m	9.74 ± 0.19 jk	0.103 fg	58.43 ± 1.12 jk	136.43 ± 1.64 k–m
A27	**25.85 ± 0.35 a**	**0.039 r**	**155.08 ± 2.09 a**	17.72 ± 0.27 b	0.056 op	106.29 ± 1.59 b	129.05 ± 0.29 no
A28	12.84 ± 0.19 j	0.078 i–k	77.06 ± 1.13 j	13.12 ± 0.33 fg	0.076 j–l	78.69 ± 2.01 fg	125.67 ± 0.37 o–q
A29	12.90 ± 0.18 j	0.077 i–k	77.42 ± 1.06 j	11.93 ± 0.33 i	0.084 hi	71.58 ± 1.98 i	148.14 ± 0.94 fg
A30	20.24 ± 0.38 c	0.049 o–r	121.43 ± 2.28 c	13.68 ± 0.11 ef	0.073 k–m	82.06 ± 0.65 ef	141.65 ± 0.72 h–j
A31	13.21 ± 0.26 ij	0.076 i–l	79.28 ± 1.59 ij	13.13 ± 0.20 fg	0.076 j–l	78.75 ± 1.23 fg	151.23 ± 0.65 f
A32	12.98 ± 0.27 j	0.077 i–k	77.90 ± 1.61 j	13.75 ± 0.27 ef	0.073 k–m	82.50 ± 1.60 ef	183.74 ± 0.81 b
A33	10.46 ± 0.31 lm	0.096 gh	62.74 ± 1.87 lm	9.71 ± 0.17 jk	0.103 fg	58.23 ± 1.00 jk	**189.30 ± 0.41 a**
A34	8.70 ± 0.17 *n*	0.115 ef	52.22 ± 1.03 *n*	12.20 ± 0.30 i	0.082 h–j	73.21 ± 1.78 i	116.48 ± 0.46 tu
A35	10.44 ± 0.18 lm	0.096 gh	62.64 ± 1.08lm	13.25 ± 0.10 fg	0.076 j–l	79.47 ± 0.57 fg	123.76 ± 0.83 p–*r*
A36	13.93 ± 0.27 hi	0.072 j–m	83.57 ± 1.61hi	14.62 ± 0.19 d	0.068 mn	87.72 ± 1.15 d	**106.35 ± 3.44 w**

*Note:* Different letters in the same column indicate that means are significantly at *p* < 0.05, Dark colors indicate the highest value, light colors indicate the lowest value, ±: SEM. In the numbering of the regions; blue color: Aralık, red color: Karakoyunlu, purple color: Iğdır, yellow color: Tuzluca and green color: represents the commercial variety.

Although Antiradical Activity (AR) values differed among regions, the highest statistically significant AR value was found in samples from region A15, while the lowest was from region A27. Inversely proportional to IC50 values, the highest average AR value by city was recorded in samples from Iğdır, whereas the lowest was observed in samples from the commercial cultivar (Table 4).

When DPPH % scavenging values were considered, results similar to IC50 were obtained. Statistically, the highest % scavenging activity was found in samples from region A27, and the lowest in samples from region A15. Similarly to IC50, the highest average IC50 value by city was found in samples from Aralık, and the lowest in samples from Iğdır (Table 4).

Although ABTS IC50 values varied by region, the highest value was found in region A11 (20.88 μg μL^−1^), while the lowest was detected in regions A15 and A18 (5.15 μg μL^−1^). On average, the highest IC50 values were observed in the commercial variety, and the lowest were found in the spear samples collected from the city of Iğdır. The highest AR value was observed in region A15, and the lowest in region A11. Among cities, the highest average AR value was found in Iğdır, while the lowest was observed in the commercial cultivar (Table 4).

When ABTS % scavenging values were evaluated, results similar to those of IC50 were obtained. Statistically, the highest % scavenging value was detected in samples from region A11, while the lowest was found in samples from regions A15 and A18. The highest average IC50 values were observed in the commercial cultivar, while the lowest were found in samples collected from Iğdır (Table 4).

The ascorbic acid content in the spear samples ranged between 189.30 and 106.35 μL L^−1^. Statistically, the highest ascorbic acid content was found in region A33, and the lowest in region A36 (commercial cultivar). Among cities, the highest average ascorbic acid content was observed in Tuzluca, and the lowest in the commercial cultivar (Table 4).

### 3.4 Determination of Free Amino Acid Levels

According to amino acid analyses performed using the LC–MS/MS system, histidine, cystine, and methionine were not detected in any of the asparagus spear samples. The amino acids lysine, arginine, and tyrosine were detected in some regions, while they were undetectable in others. The amino acids glycine, serine, asparagine, threonine, aspartic acid, glutamic acid, proline, valine, isoleucine, leucine, and phenylalanine were detected in all regions (Table 5).

**TABLE 5:** Free amino acid contents in asparagus spears (mg/100 g).

Region	Lysine	Arginine	Glycine	Serine	Asparagine	Threonine	Aspartic acid	Glutamic acid	Proline	Valine	Tyrosine	Isoleucine	Leucine	Phenylalanine
A1	**257.74 ± 18.7**	**1004.97 ± 85.4**	197.64 ± 21.4	515.83 ± 46.2	**3075.15 ± 329.0**	231.96 ± 10.6	175.81 ± 11.0	**683.77 ± 42.5**	1268.38 ± 120.1	265.90 ± 25.0	**189.43 ± 16.2**	92.73 ± 7.3	165.48 ± 7.4	117.89 ± 10.9
A2	56.78 ± 4.1	122.03 ± 10.4	137.67 ± 14.9	235.17 ± 21.0	1641.52 ± 175.6	73.85 ± 3.4	37.68 ± 2.4	134.71 ± 8.4	263.39 ± 24.9	112.42 ± 10.6	ND	72.41 ± 5.7	87.79 ± 4.0	81.86 ± 7.6
A3	30.62 ± 2.2	126.52 ± 10.8	77.84 ± 8.4	331.41 ± 29.7	2146.96 ± 229.7	145.14 ± 6.6	117.20 ± 7.3	158.27 ± 9.8	157.47 ± 14.9	147.48 ± 13.9	22.26 ± 1.9	63.11 ± 4.9	82.74 ± 3.7	82.68 ± 7.6
A4	75.38 ± 5.5	372.74 ± 31.7	208.44 ± 22.6	457.17 ± 40.9	2387.52 ± 255.5	147.77 ± 6.8	102.43 ± 6.4	466.16 ± 29.0	509.39 ± 48.2	271.84 ± 25.6	65.85 ± 5.6	80.60 ± 6.3	112.68 ± 5.1	99.41 ± 9.2
A5	22.95 ± 1.7	73.77 ± 6.3	80.68 ± 8.7	322.58 ± 28.9	821.72 ± 87.9	85.27 ± 3.9	68.70 ± 4.3	248.25 ± 15.4	124.01 ± 11.7	160.85 ± 15.2	15.12 ± 1.3	61.38 ± 4.8	81.77 ± 3.7	96.15 ± 8.9
A6	74.13 ± 5.4	288.96 ± 24.6	241.06 ± 26.1	602.16 ± 53.9	1592.46 ± 170.4	155.42 ± 7.1	142.10 ± 8.9	263.37 ± 16.4	1704.85 ± 161.4	372.84 ± 35.1	69.25 ± 5.9	81.70 ± 6.4	128.22 ± 5.8	106.23 ± 9.8
A7	6.46 ± 0.5	67.54 ± 5.7	118.08 ± 12.8	314.11 ± 28.1	1303.94 ± 139.5	109.40 ± 5.0	67.46 ± 4.2	242.66 ± 15.1	689.36 ± 65.3	188.31 ± 17.7	51.38 ± 4.4	71.10 ± 5.6	95.81 ± 4.3	91.58 ± 8.5
A8	49.53 ± 3.6	205.50 ± 17.5	171.97 ± 18.6	500.58 ± 44.8	1045.00 ± 111.8	130.11 ± 6.0	116.70 ± 7.3	274.98 ± 17.1	964.62 ± 91.3	283.37 ± 26.7	56.18 ± 4.8	67.12 ± 5.2	96.12 ± 4.3	102.45 ± 9.5
A9	64.57 ± 4.7	760.01 ± 64.6	314.62 ± 34.1	**821.49 ± 73.5**	2587.90 ± 276.9	195.16 ± 8.9	182.66 ± 11.4	297.35 ± 18.5	1274.95 ± 120.7	366.62 ± 34.5	70.63 ± 6.0	87.13 ± 6.8	189.56 ± 8.5	**151.14 ± 14.0**
A10	50.14 ± 3.6	345.40 ± 29.4	137.55 ± 14.9	580.27 ± 51.9	1519.67 ± 162.6	187.01 ± 8.6	125.69 ± 7.9	363.11 ± 22.6	1739.42 ± 164.7	**514.98 ± 48.5**	99.68 ± 8.5	90.48 ± 7.1	168.27 ± 7.6	131.78 ± 12.2
A11	64.04 ± 4.6	401.15 ± 34.1	262.27 ± 28.4	453.76 ± 40.6	1320.88 ± 141.3	**239.32 ± 11.0**	139.81 ± 8.7	350.13 ± 21.8	**1792.80 ± 169.8**	228.92 ± 21.6	16.12 ± 1.4	53.24 ± 4.2	90.39 ± 4.1	88.32 ± 8.2
A12	40.41 ± 2.9	453.23 ± 38.5	231.66 ± 25.1	409.83 ± 36.7	1096.13 ± 117.3	180.55 ± 8.3	110.86 ± 6.9	230.47 ± 14.3	1678.20 ± 158.9	410.79 ± 38.7	131.30 ± 11.2	99.28 ± 7.8	198.43 ± 8.9	158.45 ± 14.6
A13	60.40 ± 4.4	182.37 ± 15.5	205.71 ± 22.3	350.51 ± 31.4	1594.46 ± 170.6	155.35 ± 7.1	77.89 ± 4.9	354.86 ± 22.1	1064.79 ± 100.8	329.98 ± 31.1	42.79 ± 3.7	79.02 ± 6.2	154.06 ± 6.9	113.66 ± 10.5
A14	29.99 ± 2.2	ND	110.73 ± 12.0	225.98 ± 20.2	802.49 ± 85.9	107.39 ± 4.9	85.29 ± 5.3	177.98 ± 11.1	592.30 ± 56.1	210.04 ± 19.8	ND	53.26 ± 4.2	71.71 ± 3.2	70.04 ± 6.5
A15	ND	ND	**27.04 ± 2.9**	173.91 ± 15.6	**113.76 ± 12.2**	**53.00 ± 2.4**	37.40 ± 2.3	98.93 ± 6.2	244.06 ± 23.1	**88.11 ± 8.3**	ND	**43.14 ± 3.4**	**42.67 ± 1.9**	**54.04 ± 5.0**
A16	13.12 ± 1.0	ND	133.38 ± 14.4	323.85 ± 29.0	756.09 ± 80.9	116.57 ± 5.3	41.37 ± 2.6	150.56 ± 9.4	572.65 ± 54.2	170.98 ± 16.1	ND	56.20 ± 4.4	62.40 ± 2.8	73.28 ± 6.8
A17	26.90 ± 2.0	210.71 ± 17.9	165.65 ± 17.9	202.55 ± 18.1	623.01 ± 66.7	73.62 ± 3.4	35.31 ± 2.2	163.73 ± 10.2	416.57 ± 39.4	176.12 ± 16.6	14.06 ± 1.2	52.91 ± 4.1	64.70 ± 2.9	64.10 ± 5.9
A18	**1.11 ± 0.1**	307.90 ± 26.2	147.51 ± 16.0	319.33 ± 28.6	517.89 ± 55.4	108.33 ± 5.0	55.82 ± 3.5	274.31 ± 17.1	900.53 ± 85.3	169.77 ± 16.0	ND	58.06 ± 4.5	63.54 ± 2.9	70.74 ± 6.5
A19	21.14 ± 1.5	97.62 ± 8.3	89.72 ± 9.7	320.25 ± 28.7	792.54 ± 84.8	99.37 ± 4.6	31.46 ± 2.0	190.24 ± 11.8	196.57 ± 18.6	158.48 ± 14.9	ND	48.86 ± 3.8	62.27 ± 2.8	72.01 ± 6.6
A20	21.89 ± 1.6	**1.63 ± 0.1**	79.76 ± 8.6	**163.57 ± 14.6**	919.38 ± 98.4	74.81 ± 3.4	42.62 ± 2.7	119.70 ± 7.4	**61.95 ± 5.9**	109.79 ± 10.3	ND	85.21 ± 6.7	71.80 ± 3.2	67.66 ± 6.2
A21	64.55 ± 4.7	59.80 ± 5.1	178.61 ± 19.3	304.75 ± 27.3	999.04 ± 106.9	112.32 ± 5.1	**195.97 ± 12.2**	149.41 ± 9.3	74.88 ± 7.1	225.25 ± 21.2	24.97 ± 2.1	85.97 ± 6.7	82.26 ± 3.7	74.28 ± 6.9
A22	30.22 ± 2.2	ND	168.02 ± 18.2	283.38 ± 25.4	879.13 ± 94.1	89.07 ± 4.1	54.58 ± 3.4	152.81 ± 9.5	651.19 ± 61.7	149.83 ± 14.1	11.64 ± 1.0	55.91 ± 4.4	63.41 ± 2.9	75.97 ± 7.0
A23	43.99 ± 3.2	ND	33.23 ± 3.6	290.59 ± 26.0	544.48 ± 58.3	119.95 ± 5.5	43.31 ± 2.7	107.07 ± 6.7	343.32 ± 32.5	130.25 ± 12.3	36.60 ± 3.1	52.93 ± 4.1	54.34 ± 2.4	61.48 ± 5.7
A24	54.55 ± 4.0	23.06 ± 2.0	191.12 ± 20.7	336.44 ± 30.1	1652.54 ± 176.8	151.46 ± 6.9	124.27 ± 7.8	173.92 ± 10.8	549.33 ± 52.0	360.07 ± 33.9	17.96 ± 1.5	122.50 ± 9.6	122.25 ± 5.5	89.90 ± 8.3
A25	27.81 ± 2.0	ND	52.26 ± 5.7	307.81 ± 27.5	673.93 ± 72.1	64.01 ± 2.9	41.53 ± 2.6	100.28 ± 6.2	661.68 ± 62.7	201.32 ± 19.0	ND	62.77 ± 4.9	71.89 ± 3.2	66.16 ± 6.1
A26	10.45 ± 0.8	ND	88.68 ± 9.6	367.22 ± 32.9	555.13 ± 59.4	108.11 ± 5.0	47.86 ± 3.0	188.09 ± 11.7	728.13 ± 69.0	165.61 ± 15.6	17.19 ± 1.5	60.88 ± 4.8	61.45 ± 2.8	70.27 ± 6.5
A27	23.17 ± 1.7	91.74 ± 7.8	119.49 ± 12.9	357.82 ± 32.0	941.94 ± 100.8	92.22 ± 4.2	57.06 ± 3.6	151.38 ± 9.4	543.50 ± 51.5	150.52 ± 14.2	7.17 ± 0.6	47.76 ± 3.7	55.31 ± 2.5	59.99 ± 5.5
A28	54.79 ± 4.0	ND	139.53 ± 15.1	375.38 ± 33.6	750.34 ± 80.3	81.07 ± 3.7	42.97 ± 2.7	139.05 ± 8.6	729.84 ± 69.1	184.16 ± 17.3	7.97 ± 0.7	57.56 ± 4.5	79.29 ± 3.6	98.45 ± 9.1
A29	13.12 ± 1.0	77.52 ± 6.6	186.43 ± 20.2	260.41 ± 23.3	1320.40 ± 141.3	104.02 ± 4.8	111.86 ± 7.0	302.55 ± 18.8	1265.28 ± 119.8	236.41 ± 22.3	14.18 ± 1.2	77.52 ± 6.1	84.39 ± 3.8	89.19 ± 8.2
A30	36.49 ± 2.6	ND	237.17 ± 25.7	256.97 ± 23.0	1019.79 ± 109.1	109.94 ± 5.0	58.62 ± 3.7	331.45 ± 20.6	673.15 ± 63.7	209.16 ± 19.7	53.83 ± 4.6	81.59 ± 6.4	92.43 ± 4.2	77.44 ± 7.1
A31	10.49 ± 0.8	ND	90.76 ± 9.8	316.46 ± 28.3	423.59 ± 45.3	85.71 ± 3.9	34.18 ± 2.1	**87.91 ± 5.5**	496.75 ± 47.0	128.56 ± 12.1	17.20 ± 1.5	60.37 ± 4.7	62.95 ± 2.8	66.00 ± 6.1
A32	48.21 ± 3.5	33.14 ± 2.8	171.28 ± 18.5	386.66 ± 34.6	1088.42 ± 116.5	114.82 ± 5.3	58.65 ± 3.7	160.93 ± 10.0	863.23 ± 81.7	285.24 ± 26.9	15.82 ± 1.4	117.25 ± 9.2	95.00 ± 4.3	78.06 ± 7.2
A33	ND	ND	80.76 ± 8.7	194.23 ± 17.4	731.53 ± 78.3	91.30 ± 4.2	44.11 ± 2.8	107.57 ± 6.7	514.43 ± 48.7	135.64 ± 12.8	**0.98 ± 0.1**	52.25 ± 4.1	58.71 ± 2.6	65.03 ± 6.0
A34	12.65 ± 0.9	47.05 ± 4.0	96.78 ± 10.5	246.88 ± 22.1	962.39 ± 103.0	105.89 ± 4.8	74.63 ± 4.7	125.30 ± 7.8	214.52 ± 20.3	158.33 ± 14.9	5.23 ± 0.4	67.94 ± 5.3	71.59 ± 3.2	70.71 ± 6.5
A35	16.08 ± 1.2	47.85 ± 4.1	166.35 ± 18.0	239.42 ± 21.4	652.96 ± 69.9	88.01 ± 4.0	**24.87 ± 1.6**	126.96 ± 7.9	783.76 ± 74.2	151.93 ± 14.3	22.17 ± 1.9	52.69 ± 4.1	52.38 ± 2.4	68.02 ± 6.3
A36	138.80 ± 10.1	66.05 ± 5.6	**598.43 ± 64.8**	267.69 ± 24.0	1667.77 ± 178.5	214.74 ± 9.8	115.23 ± 7.2	185.03 ± 11.5	152.82 ± 14.5	433.38 ± 40.8	37.70 ± 3.2	**246.41 ± 19.3**	**235.52 ± 10.6**	142.34 ± 13.1

*Note:* ±: SEM. Dark colors indicate the highest value, light colors indicate the lowest value. In the numbering of the regions; blue color: Aralık, red color: Karakoyunlu, purple color: Iğdır, yellow color: Tuzluca and green color: represents the commercial variety.

Abbreviation: ND, Not Detected.

Lysine content ranged from 1.11 to 257.74 mg/100 g across regions. The highest lysine content was found in region A1, while the lowest was observed in region A18. On average, the highest lysine content was recorded in the commercial cultivar, whereas the lowest city‐wide average was calculated for Karakoyunlu (Table 5).

Arginine content was highest in region A1 with 1004.97 mg/100 g, and lowest in region A20 with 1.63 mg/100 g. Among the cities, the highest average arginine content was found in Aralık, while the lowest was observed in the commercial cultivar (Table 5).

Glycine content reached a maximum of 598.43 mg/100 g in region A36 (commercial cultivar), while the lowest value was 27.04 mg/100 g in region A15. The commercial cultivar showed the highest average glycine content, whereas the city with the lowest average was Iğdır (Table 5).

Serine content was highest in region A9 with 821.49 and lowest in region A20 with 163.57 mg/100 g. The highest average serine content was observed in Aralık, while the lowest was found in Iğdır (Table 5).

Serine content was highest in region A9 with 821.49 mg/100 g, while the lowest was recorded in region A20 with 163.57 mg/100 g. Among the cities, the highest average serine content was found in Aralık, whereas the lowest was observed in Iğdır (Table 5).

Asparagine levels varied between 113.76 and 3075.15 mg/100 g across the regions. The highest asparagine content was detected in region A1, and the lowest in region A15. On a city basis, the highest average asparagine content was observed in Aralık, while the lowest was found in Tuzluca (Table 5).

Threonine content was highest in region A11 with 239.32 mg/100 g, and lowest in region A15 with 53.00 mg/100 g. The highest average threonine content was recorded in the commercial cultivar, while the lowest citywide average was observed in Tuzluca (Table 5).

Aspartic acid levels varied across regions, with the highest content found in region A21 (195.97 mg/100 g) and the lowest in region A35 (24.87 mg/100 g). The commercial cultivar had the highest average aspartic acid content, while the lowest citywide average was calculated for Tuzluca (Table 5).

Glutamic acid content reached a maximum of 683.77 and a minimum of 87.91 mg/100 g in region A1 and region A31, respectively. The highest average glutamic acid content was found in Aralık, while the lowest was observed in Iğdır (Table 5).

The highest proline content was detected in region A11 with 1792.80 mg/100 g, while the lowest was found in region A20 with 61.95 mg/100 g. Among cities, the highest average proline content was found in Karakoyunlu, while the lowest was observed in the commercial cultivar (Table 5).

Valine contents were observed to vary between 88.11 and 514.98 mg/100 g across different regions. The highest valine content was found in region A10, and the lowest in region A15. While the highest average valine content was detected in the commercial cultivar, the lowest average valine content among cities was found in Iğdır (Table 5).

Although tyrosine contents varied by region, the highest was 189.43 mg/100 g in region A1, and the lowest was 0.98 mg/100 g in region A33. Among cities, the highest average tyrosine content was recorded in Aralık, while the lowest was found in Iğdır (Table 5).

In asparagus spears, varying results were obtained for leucine and isoleucine contents depending on the region. However, the highest contents of both amino acids were calculated in region A36 (Commercial cultivar) as 235.52 and 246.41 mg/100 g, respectively, while the lowest contents were found in region A15 as 42.67 and 43.14 mg/100 g, respectively (Table 5).

Phenylalanine contents varied by region; the highest was 151.14 mg/100 g in region A9, and the lowest was 54.04 mg/100 g in region A15. While the highest average phenylalanine content was found in the commercial cultivar, the lowest average content among cities was observed in Iğdır (Table 5).

### 3.5 Determination of Diosgenin Amount and Total Saponin Content

Diosgenin contents in asparagus spears varied between 0.872 and 0.008 mg/g depending on the region. The highest diosgenin content was detected in region A1, while the lowest was found in region A27. Among cities, the highest average diosgenin content was observed in Aralık, whereas the lowest average diosgenin content was determined in the commercial variety (Table 6).

**TABLE 6:** Diosgenin content and total saponin content equivalent to steroidal diosgenin.

Region	Diosgenin (mg g^−1^ FW)	Total saponins (mg g^−1^ FW)	Region	Diosgenin (mg g^−1^ FW)	Total saponins (mg g^−1^ FW)	Region	Diosgenin (mg g^−1^ FW)	Total saponins (mg g^−1^ FW)
A1	**0.872 ± 0.0113**	7.65 ± 0.30 m–o	A13	0.023 ± 0.0003	8.55 ± 0.34 j–m	A25	0.016 ± 0.0002	9.61 ± 0.32 f–h
A2	0.053 ± 0.0007	5.87 ± 0.12 r–t	A14	0.009 ± 0.0001	11.18 ± 0.25 b–d	A26	0.010 ± 0.0001	11.90 ± 0.62 b
A3	0.026 ± 0.0003	7.94 ± 0.32 l–n	A15	0.009 ± 0.0001	9.77 ± 0.62 fg	A27	**0.008 ± 0.0001**	9.36 ± 0.56 g–k
A4	0.027 ± 0.0004	5.43 ± 0.15 st	A16	0.015 ± 0.0002	8.44 ± 0.08 k–m	A28	0.041 ± 0.0005	8.02 ± 0.14 l–n
A5	0.034 ± 0.0004	4.06 ± 0.11 v	A17	0.012 ± 0.0002	9.87 ± 0.17 e–g	A29	0.018 ± 0.0002	7.49 ± 0.07 no
A6	0.056 ± 0.0007	**13.25 ± 0.11 a**	A18	0.017 ± 0.0002	6.82 ± 0.18 o–q	A30	0.034 ± 0.0004	6.21 ± 0.01 q–s
A7	0.242 ± 0.0031	8.76 ± 0.11 h–l	A19	0.014 ± 0.0002	6.56 ± 0.05 p–r	A31	0.056 ± 0.0007	9.39 ± 0.55 g–j
A8	0.783 ± 0.0102	10.68 ± 0.04 c–e	A20	0.020 ± 0.0003	4.41 ± 0.03 uv	A32	0.300 ± 0.0039	11.51 ± 0.07 bc
A9	0.066 ± 0.0009	9.30 ± 0.07 g–k	A21	0.019 ± 0.0002	4.18 ± 0.07 v	A33	0.033 ± 0.0004	7.23 ± 0.78 n–p
A10	0.015 ± 0.0002	10.97 ± 0.10 cd	A22	0.014 ± 0.0002	9.52 ± 0.22g–i	A34	0.038 ± 0.0005	5.08 ± 0.02 tu
A11	0.500 ± 0.0065	9.27 ± 0.09 g–k	A23	0.239 ± 0.0031	10.45 ± 0.24 d–f	A35	0.022 ± 0.0003	8.49 ± 0.12 j–m
A12	0.009 ± 0.0001	11.53 ± 0.33 bc	A24	0.020 ± 0.0003	8.65 ± 0.07 i–l	A36	0.014 ± 0.0002	**1.76 ± 0.03 w**

*Note:* Different letters in the same column indicate that means are significantly at *p* < 0.05, Dark colors indicate the highest value, light colors indicate the lowest value, ±: SEM. In the numbering of the regions; blue color: Aralık, red color: Karakoyunlu, purple color: Iğdır, yellow color: Tuzluca and green color: represents the commercial variety.

When examining the total steroidal saponin contents based on diosgenin, variations were observed among regions, ranging from 1.76 to 13.25 mg/g. Statistically, the highest total saponin content was found in region A6, while the lowest was determined in region A36 (commercial variety) (Table 6). Among cities, the highest average total saponin content was observed in Karakoyunlu, whereas the lowest average total saponin content was detected in the commercial cultivar (Table 6).

### 3.6 Correlation Analysis

**FIGURE 2:**
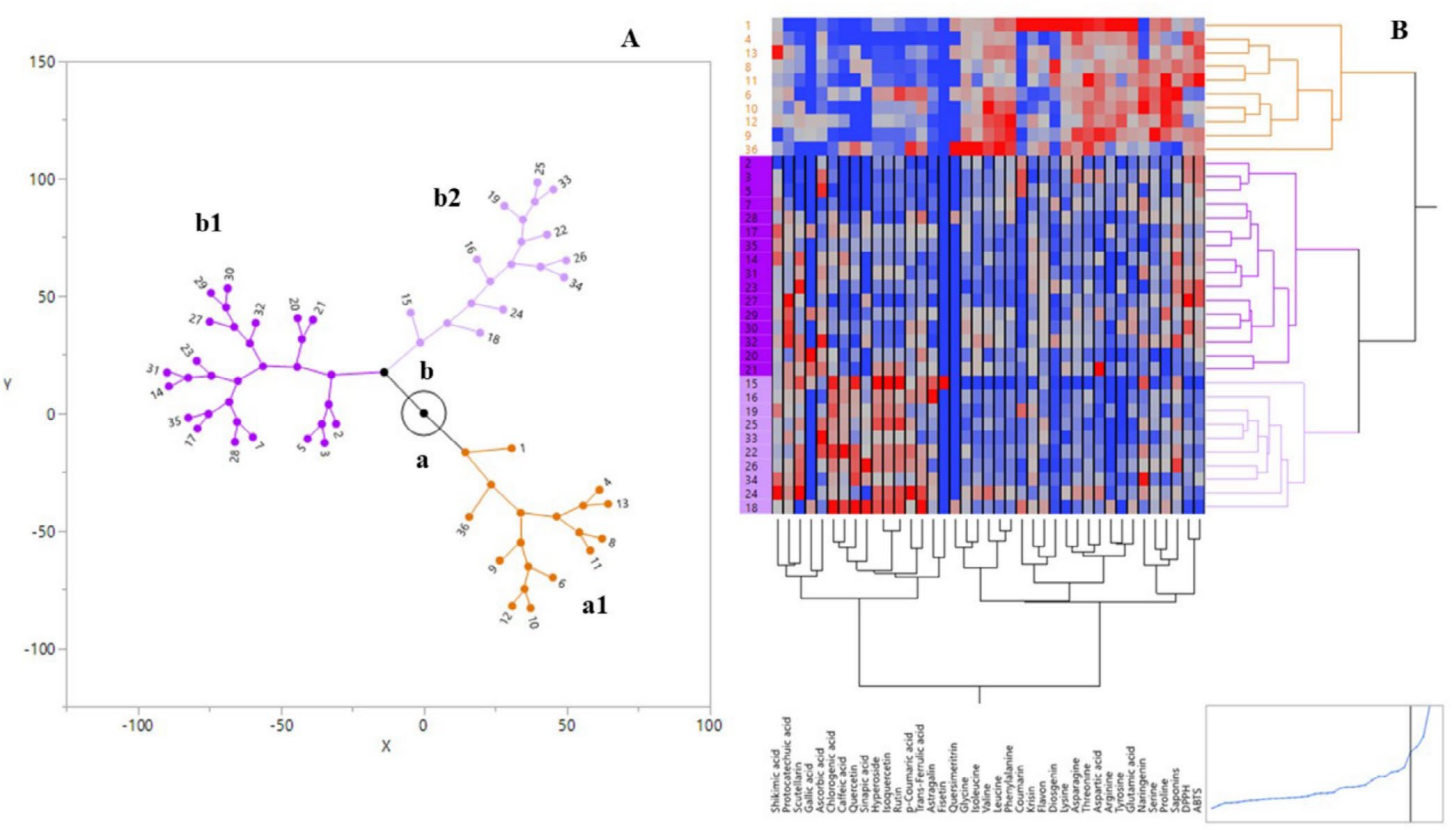
Cluster constellation plot (A) and heat map (B) of asparagus groups. See Table 1 for definition of abbreviations.

Due to the large number of locations and associated observations in our study, cluster constellation (star diagram), principal component analysis (PCA), and heat map clustering analyses were conducted to visualize, clarify, and associate the observations corresponding to each location. The clustering distance used in the analyses was determined based on correlations derived from the transformed mean values of the observations. In this context, a cluster constellation analysis was carried out to investigate the biochemical and antioxidative diversity of asparagus genotypes. According to the constellation graph analysis, the asparagus genotypes examined in 35 locations and one commercial sample were evaluated based on their biochemical properties. Based on the data analyzed, the asparagus genotypes were grouped into two main clusters and three sub‐clusters (A; A1 and B; B1, B2). The findings showed that samples from Aralık and Karakoyunlu cities, as well as the commercial variety, were mostly grouped in cluster A1 (orange), while samples from Iğdır city were predominantly in cluster B2 (light purple), and most of the samples from Tuzluca city along with samples 2, 3, and 5 from Aralık city were grouped in cluster B1 (dark purple). All purple‐colored varieties were clustered within region B (Figure 2A). The constellation graph places individuals at extreme points based on various parameters, and each cluster converges at a new point through lines representing membership (Kumari et al. 2017). The fact that the samples from Aralık and Karakoyunlu are clustered together with the commercial variety strengthens the possibility that these samples are more closely related (Figure 2A).

In addition to the cluster constellation analysis, a heat map was created to cluster the harvest locations corresponding to the investigated parameters, since the related observations were not included in the former analysis. In the heat map, red color indicates high values of observations, while blue indicates low values. Accordingly, no distinct separation was observed. In terms of location, two main clusters containing several sub‐clusters were observed. As expected, samples collected from the same region showed higher similarity indices. In particular, the samples from Iğdır city showed largely consistent results. Antioxidants and free amino acid characteristics stood out at the first level (orange), whereas phenolic compounds were more prominent at the second level (purple) (Figure 2B).

**FIGURE 3:**
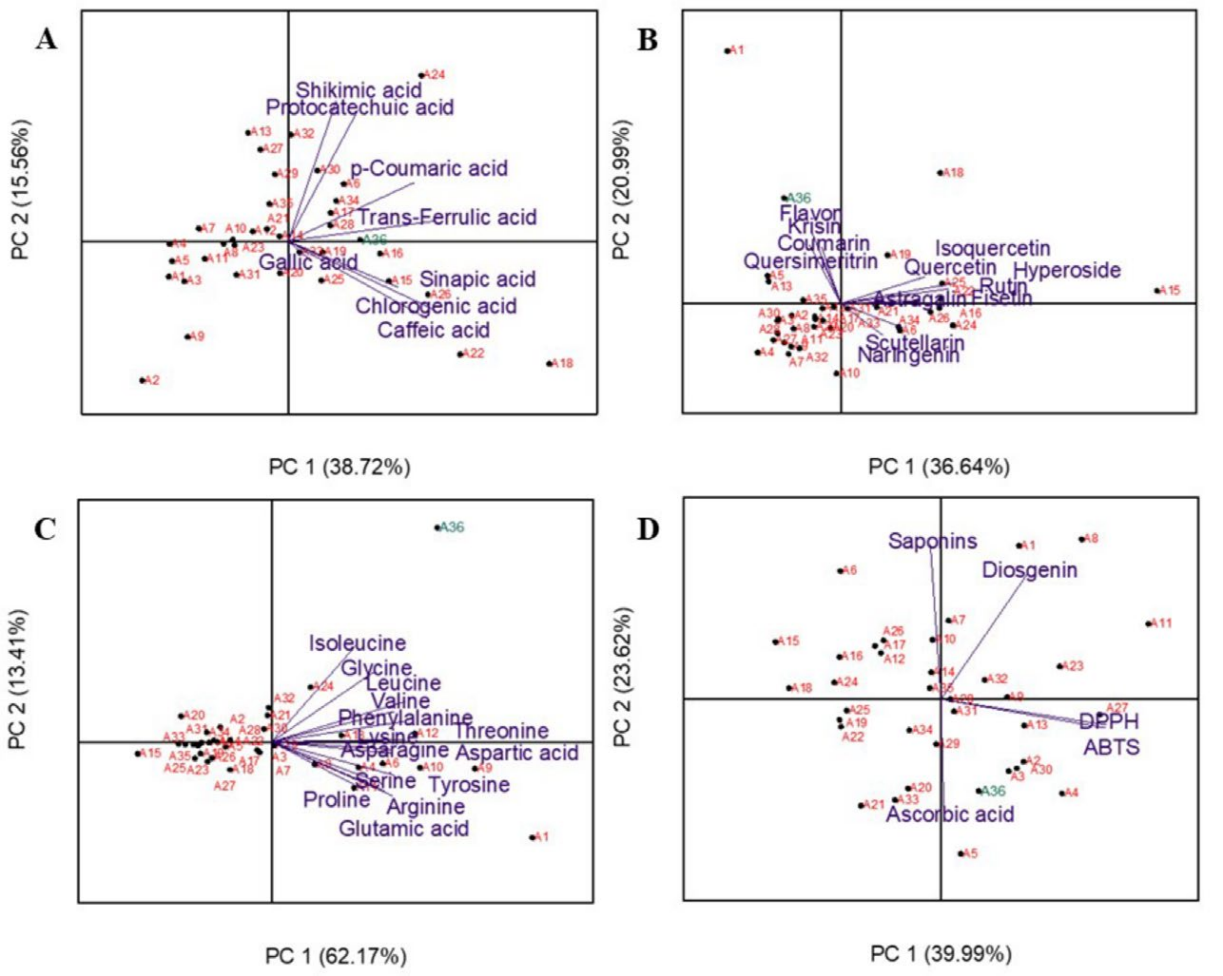
PCA for all the locations and biochemical traits: Phenolic acids (A), flavanoids (B), free amino acids (C), and antioxidants and saponins (D).

To explain the variance of observations based on locations, four principal component analyses (PCA) were conducted. The first PCA biplot was constructed using the phenolic acid values presented in Table 2, and it was found that the eigenvalues of the 36 variables included in the analysis were greater than 1. It is important to note that the first principal component (PC1) explained 38.72% of the variation observed in the phenolic acid traits of asparagus spears. In addition, the second component (PC2) accounted for 15.56% of the variation, and together, these two components explained 53.28% of the total variance. Regarding factor loadings, PC1 was mainly composed of Gallic acid, Chlorogenic acid, Caffeic acid, *p*‐Coumaric acid, Trans‐Ferulic acid, and Sinapic acid, while PC2 was mainly associated with Shikimic acid and Protocatechuic acid (Figure 3A).

The second PCA was performed based on the flavonol characteristics of the regions, and it was again found that all 36 variables had eigenvalues greater than 1. This analysis explained 57.63% of the total variance. Regarding the factor loadings, PC1 was composed of Scutellarin, Hyperoside, Rutin, Isoquercetin, Astragalin, Fisetin, Quercetin, and Naringenin, whereas PC2 consisted of Quercimeritrin, Coumarin, Chrysin, and Flavone parameters (Figure 3B).

The third PCA was related to the free amino acid characteristics of the regions, and it was found that all 36 variables included in the analysis had eigenvalues greater than 1. This PCA explained 75.58% of the total variance. Regarding the factor loadings, the amino acids Glycine and Isoleucine were associated with PC2, while the remaining amino acids contributed to PC1 (Figure 3C).

The fourth PCA was conducted based on antioxidant capacity and saponin properties, explaining 63.61% of the total variance. In terms of factor loadings, PC1 consisted of the DPPH, ABTS, and ascorbic acid parameters, while PC2 was composed of diosgenin and total saponin content (Figure 3D).

In terms of phenolic acids, wild asparagus samples were distributed across different planes, whereas the commercial variety was located centrally among all regions. For flavonoids, the commercial variety and samples from Karakoyunlu were positioned on a similar plane, while samples from other cities occupied a separate plane. In the distribution of free amino acids, the commercial variety formed a distinct group compared to the wild asparagus samples. Additionally, saponin and diosgenin parameters contributed to the separation of the commercial variety into a separate group (Figure 3).

## 4 Discussion

Along the Aras River, which stretches across the Türkiye‐Azerbaijan‐Iran‐Armenia borders in the Iğdır Plain, wild edible Asparagus spears are found in high density (Özden et al. 2023). These perennial asparagus plants have undergone natural hybridizations over the years, resulting in distinct characteristics between populations from different cities. It is believed that environmental conditions in the region, the age of the asparagus plants, their inherent botanical traits, and potential interactions between these factors are the main determinants of asparagus quality and nutritional content. For instance, the phenolic compound profiles of samples collected from different areas may vary significantly, which can influence the total antioxidant capacity, saponin, or amino acid contents. Some of these traits—such as antioxidant properties or phenolic contents—may be subject to genotype × environment interactions, leading to variations in antioxidant performance across locations (Drinkwater et al. 2014). These differences may be affected by environmental factors such as altitude and temperature in the sampling regions, causing significant changes in the bioactive composition and quality of the wild asparagus. Therefore, in our study, we analyzed both each individual sampling site and the average values at the city level to better understand these variations.

Our study revealed that wild asparagus spears, particularly those growing along the riverbanks of the Iğdır region, exhibit substantial biochemical diversity (Figure 2; Figure 3). When compared with both the analytical results of commercial cultivars and previously reported data on wild asparagus, the samples collected from Iğdır generally demonstrated higher levels of phenolic compounds. Compared to the wild asparagus spears examined by Kobus‐Cisowska et al. (2019), the spears collected from the Iğdır Plain had lower levels of gallic acid but significantly higher levels of protocatechuic acid, caffeic acid, chlorogenic acid, *p*‐coumaric acid, ferulic acid, and sinapic acid. Similarly, when compared with the flavonol contents reported in the same study, the levels of astragalin in the Iğdır samples were lower, while the contents of rutin, isoquercetin, hyperoside, and quercetin were higher. Gao et al. (2024) reported that polyphenol content in *A. officinalis* genotypes ranged between 8.67 and 6.34 mg/g DW, and flavonoid contents ranged from 8.22 to 4.21 mg/g DW. While our findings generally fall within these ranges, some regions in the Iğdır Plain exhibited significantly higher values. Furthermore, although the concentrations of certain individual phenolic acids and flavonols were lower in some regional samples compared to the commercial cultivar, the total phenolic acid and total flavonol contents were consistently higher in all wild samples. In particular, samples from central Iğdır and Tuzluca exhibited higher average levels of phenolic compounds and flavonoids (Tables 2 and 3).

It is particularly important to emphasize the high rutin content observed in the asparagus samples analyzed from the Iğdır Plain. Rutin is one of the primary functional compounds found in asparagus (Chin et al. 2002; Maeda et al. 2005) and exhibits biological activities in humans, such as mitigating capillary fragility (Griffith et al. 1944). The antioxidant capacity of green asparagus is primarily attributed to rutin (quercetin‐3‐O‐rutinoside), a flavonoid derivative of quercetin (Siomos 2018), which constitutes the main polyphenolic compound in green asparagus spears (Motoki et al. 2012; Soteriou et al. 2021). Based on these findings, asparagus can be promoted as a rich source of rutin, and cultivation techniques may be developed in the future to enhance rutin content (Motoki 2019). Drinkwater et al. (2014) reported that rutin accumulates at higher levels compared to other phenolic compounds in asparagus spears, is predominantly regulated genetically, and can vary widely among cultivars, ranging from 0.2 to 15 mg/g DW. In this context, the naturally growing asparagus in the Iğdır Plain, with its high rutin content, may represent a significant potential antioxidant source (Table 3).

In recent years, there has been a growing interest in wild edible plants in modern societies due to their nutritional and pharmaceutical properties, and their potential as sources of unique flavors (Ogle et al. 2004; Tardio et al. 2006; Barucha and Pretty 2010; Sanchez‐Mata et al. 2011; Jaramillo‐Carmona et al. 2017; Kaska et al. 2018). Today, wild asparagus species are gaining increasing scientific attention not only for their nutritional value but also for their richness in bioactive compounds associated with health benefits. Carotenoids, phenolics, saponins, ascorbic acid, and other organic acids have become focal points of this interest (Guillén et al. 2008; Sanchez‐Mata et al. 2011; Morales et al. 2012; Tardio et al. 2016). Several recent studies have shown that wild asparagus has higher contents of phenolic and fatty acids compared to cultivated varieties (Guillén et al. 2008; Morales et al. 2012). Our findings also demonstrate that the asparagus spears collected from the Iğdır region are particularly rich in compounds such as caffeic acid, chlorogenic acid, rutin, and hyperoside (Table 4), indicating that these plants possess high antioxidant capacity.

Total polyphenol content is closely associated with DPPH free radical scavenging capacity (Maeda et al. 2005). Therefore, polyphenols are suitable markers to characterize the radical scavenging ability of asparagus. The inhibition of free radicals by green asparagus is supported by various naturally occurring polyphenols, including rutin and quercetin (Fuentes‐Alventosa et al. 2009; Kobus‐Cisowska et al. 2019). In our study, DPPH and ABTS assays were conducted to evaluate the antioxidant capacity of asparagus spears, and when individual regions were examined, variations were observed both within the regions and in comparison with the commercial cultivar. However, when the averages of all 35 regions were considered, the DPPH IC50 values were found to be lower than that of the commercial cultivar, and a similar trend was observed for ABTS IC50 values as well.

Interestingly, when considering the total antioxidant capacity—i.e., the sum of DPPH and ABTS IC50 values—the average values of samples collected from the city of Aralık were higher than those of the commercial cultivar. Additionally, the total antioxidant capacity of purple asparagus spears coded A32 and A33 used in our study was found to be lower compared to the other regions (Figure 2B). Although the highest DPPH and ABTS activities were observed in green asparagus regions, the average values of the four purple asparagus spears included in the study were not significantly distant from those of green asparagus, indicating that their antioxidant contents are still considerable.

According to DPPH assays, green asparagus exhibits the highest antioxidant activity (Kulczyński et al. 2016). This high antioxidant activity contributes to the enhanced nutritional value of green asparagus (Kobus‐Cisowska et al. 2017). In addition to improving nutritional quality, the antioxidants present in asparagus may offer potential health benefits such as reducing the risk of cancer, cardiovascular diseases, and cerebrovascular disorders (Wang et al. 2013; Zhong et al. 2015; Palfi et al. 2017; Chileh‐Chelh et al. 2024).

A linear relationship is believed to exist between the phenolic compound content and antioxidant capacity of asparagus spears. Indeed, Soteriou et al. (2021) reported a positive correlation (*r* = 0.67, *p* < 0.01) between the DPPH antioxidant capacity and the total phenolic content in asparagus spears. Similarly, our findings revealed a noticeable association between DPPH and ABTS values and the levels of phenolic compounds and ascorbic acid. This indicates that regional wild asparagus not only possesses valuable nutritional properties but also contains health‐promoting bioactive compounds.

All regional samples exhibited higher ascorbic acid content compared to the commercial cultivar. When the regional data were averaged, the mean ascorbic acid concentration remained consistently higher than that of the commercial variety (Table 4). Previous studies by Žebrauskienė et al. (2013) and Ku et al. (2018) have reported substantial variation in vitamin C content between cultivated and wild asparagus spears. Ascorbic acid is widely recognized for its essential role in human nutrition and its contribution to maintaining health, primarily due to its function as a potent natural antioxidant (Shou et al. 2007). When assessed alongside phenolic compounds, these findings suggest that wild asparagus spears from the region possess strong antioxidant potential. This is supported by the fact that the majority of natural antioxidants are phenolic in nature, with key contributors including tocopherols, ascorbic acid, flavonoids, and phenolic acids. Therefore, wild asparagus populations in the study area may offer considerable nutritional value.

Amino acid contents of wild asparagus spears collected from different regions varied, but when the average of all 35 regions was considered, it was found that the contents of lysine, glycine, asparagine, threonine, aspartic acid, valine, isoleucine, leucine, and phenylalanine were lower compared to the commercial cultivar. In contrast, arginine, serine, glutamic acid, proline, and tyrosine contents were higher (Table 5). When the city averages were examined, the samples collected from the city of Aralık were determined to have a higher total amino acid content than both the commercial variety and the overall average of the other cities. Compared with the study by Słupski et al. (2010), which determined the amino acid contents of wild asparagus spears, the wild asparagus spears collected from the Iğdır region were found to contain lower levels of glutamic acid, aspartic acid, methionine, tyrosine, leucine, lysine, histidine, and cysteine, while higher levels of arginine, isoleucine, proline, phenylalanine, serine, valine, threonine, and glycine were observed. Additionally, according to the findings of King and O'Donoghue (1995), the asparagine content in wild asparagus spears collected from the Iğdır region was found to be higher.

Gao et al. (2024) reported that the total free amino acid content in asparagus spears ranged between 9.98 and 5.60 g/100 g DW. Based on these values, the wild asparagus from Iğdır can be considered to have a high amino acid content. Overall, asparagus is considered a high‐quality protein source as it contains most of the essential amino acids, which make up approximately 40%–43% of total amino acids. While glutamic acid and aspartic acid are found in high amounts, methionine, cysteine, and leucine are present in lower amounts (Słupski et al. 2010). These findings are consistent with our results and suggest that the regional wild asparagus may also possess high‐quality protein content.

Jaramillo‐Carmona et al. (2017) reported that the total saponin content in the wild asparagus they studied was found to be, on average, 2.2 mg/100 g fresh weight (FW). Chileh‐Chelh et al. (2024), on the other hand, observed total saponin contents ranging between 6.69 and 7.50 mg/g FW in *A. officinalis* cultivated varieties. According to our research findings, the total saponin content in wild asparagus spears collected from the Iğdır region falls within this range in some areas, while notably high values were detected in certain locations such as A6. When considering the city averages, Karakoyunlu was identified as having the highest average saponin content (Table 6). Compared to commercial cultivars, the total saponin content in all wild asparagus spears was found to be considerably higher.

Saponins present in *A. officinalis* spears are involved in various biological activities including anti‐inflammatory, antioxidant, antitumor, antifungal, and antimicrobial roles. However, since the mechanisms of action, ideal dosages, and application methods of saponins are not fully understood, further research is needed to clarify these aspects (Hamdi et al. 2021; Valdes and Stoycheva 2024). Some studies have also suggested that saponins may play a role in promoting healthy digestion and regulating blood lipid levels (Marrelli et al. 2016; Chileh‐Chelh et al. 2024). In this respect, wild asparagus from the Iğdır Plain is considered to have great potential for health benefits.

The primary effect of saponins involves their inclusion in dietary programs and their ability to induce cell death in cancer cells through various pathways (Lorent et al. 2014). The most important dietary sources of saponins are triterpenoid types obtained from legumes such as soybeans and chickpeas. Steroidal saponins, which are found in species like asparagus, are less common and their effects are more intriguing (Jaramillo‐Carmona et al. 2017). The saponin content varies depending on the climatic conditions of the environment where asparagus is grown, the plant parts sampled, the spear color, and whether it is a cultivated or wild type (Negi et al. 2011). Based on comparisons and the results obtained, it can be suggested that asparagus spears collected from the Iğdır region are rich in steroidal saponins.

When the research findings are broadly compared with existing literature, the average two‐year data of asparagus shoots collected from 35 locations in the Iğdır Plain revealed higher total saponin contents than those reported by Jaramillo‐Carmona et al. (2017). Regarding phenolic acid composition, compared to the results of Kobus‐Cisowska et al. (2019), gallic acid was found to be lower, whereas protocatechuic acid, caffeic acid, chlorogenic acid, p‐coumaric acid, ferulic acid, and sinapic acid contents were higher. In terms of flavonol composition, astragalin content was lower, while rutin, quercetin, isoquercetin, and hyperoside levels were higher than those reported by Kobus‐Cisowska et al. (2019). With respect to free amino acids, compared to the findings of Słupski et al. (2010), glutamic acid, aspartic acid, tyrosine, leucine, and lysine contents were lower, whereas arginine, isoleucine, proline, phenylalanine, serine, valine, threonine, glycine, and asparagine were found at higher levels.

In light of the data obtained, this research determined the contents of major phenolic acids, key flavonols, antioxidant capacity, ascorbic acid, free amino acids, diosgenin, and total saponins associated with diosgenin in wild asparagus spears naturally growing in 35 different regions of the Iğdır Plain. In general, it was observed that wild asparagus in the Iğdır Plain had higher contents of total phenolic compounds, amino acids, and antioxidant capacity compared to commercial varieties and other wild asparagus samples reported in the literature, making them particularly valuable in terms of nutrition and healthy consumption. The fact that vitamin C and total saponin content were found to be higher in all regions compared to the commercial sample is considered highly significant. Notably, samples collected from the regions in the central city of Iğdır yielded the most ideal results and stood out in terms of nutritional and antioxidant properties. From this perspective, the wild asparagus found in the region may be more nutritious and more easily preferred by consumers. Among the 35 regions, A1, A15, A22, A24, and A27 emerged as the most prominent based on all examined parameters. Cultivation studies through seed propagation from these regions, as well as the development of new variety candidates through single or mass selection breeding, could increase the diversity of asparagus varieties on the market and enhance their nutritional value. In future studies, it is considered necessary to continue investigations, particularly focusing on specific saponin contents, as well as sugars, alkaloids, and vitamins.

## 5 Conclusion

This study provides the first scientific data on the biochemical composition of naturally growing wild edible asparagus in the Iğdır region. The wild edible asparagus spears naturally distributed across the Iğdır Plain demonstrate a remarkable biochemical diversity and richness in health‐promoting compounds such as rutin, phenolic acids, flavonols, ascorbic acid, amino acids, and saponins. While the antioxidant capacity of some wild samples was slightly lower than that of commercial cultivars in specific assays, their overall bioactive composition indicates a high potential for nutritional and pharmaceutical applications. These findings not only contribute to our understanding of the biochemical potential of wild Asparagus spp. in semi‐arid riparian ecosystems but also underscore the importance of conserving these natural populations. Future studies should explore the domestication potential of superior genotypes and investigate optimal agronomic practices to enhance bioactive compound accumulation while ensuring the sustainable use of these valuable wild resources. Efforts toward the domestication of these species and the preservation of their nutritional potential can help prevent their disappearance due to overharvesting from natural habitats.

## Author Contributions

**Eren Özden:** conceptualization (lead), formal analysis (lead), funding acquisition (lead), methodology (lead), resources (lead), visualization (lead), writing – original draft (lead), writing – review and editing (lead). **Adnan Aydin:** data curation (equal), resources (equal), writing – review and editing (supporting). **Kaan Hürkan:** formal analysis (equal), resources (equal), software (equal), writing – review and editing (supporting). **Mehmet Nuri Atalar:** funding acquisition (equal), validation (equal). **Ayşe Türkhan:** methodology (equal), writing – review and editing (supporting).

## Conflicts of Interest

The authors declare no conflicts of interest.

## Supporting information


**Figure S1:** fsn370849‐sup‐0001‐FiguresS1‐S7.docx.

## Data Availability

All the associated data will be provided upon reasonable request by the corresponding author.
