# Correction to “Bioactive Compounds and Antioxidant Capacity of Wild Edible Asparagus in the Iğdır Plain”

**DOI:** 10.1002/fsn3.71122

**Published:** 2025-10-17

Özden E. Aydin A. Hürkan K. Atalar M. N. Türkhan A. 2025 “Bioactive Compounds and Antioxidant Capacity of Wild Edible Asparagus in the Iğdır Plain.” Food Science & Nutrition 13 e70849 10.1002/fsn3.70849

In the published version of the article, the following Acknowledgment section was missing:

Acknowledgments

This work was supported by TUBITAK (The Scientific and Technological Research Council of Türkiye) (Project number: 120O213).

We apologize for this error.

