# Supplementary material for: Bioactive Compounds and Antioxidant Capacity of Wild Edible Asparagus in the Iğdır Plain
Source: Food Sci Nutr. 2025 Aug 28;13(9):e70849. doi: 10.1002/fsn3.70849 (PMC12394889; doi:10.1002/fsn3.70849)
Supplement: Supplementary file 1 — Figure S1: fsn370849‐sup‐0001‐FiguresS1‐S7.docx. [file FSN3-13-e70849-s001.docx]

**Supplementary Data**

**Bioactive Compounds and Antioxidant Capacity of Wild Edible Asparagus in the Iğdır Plain**


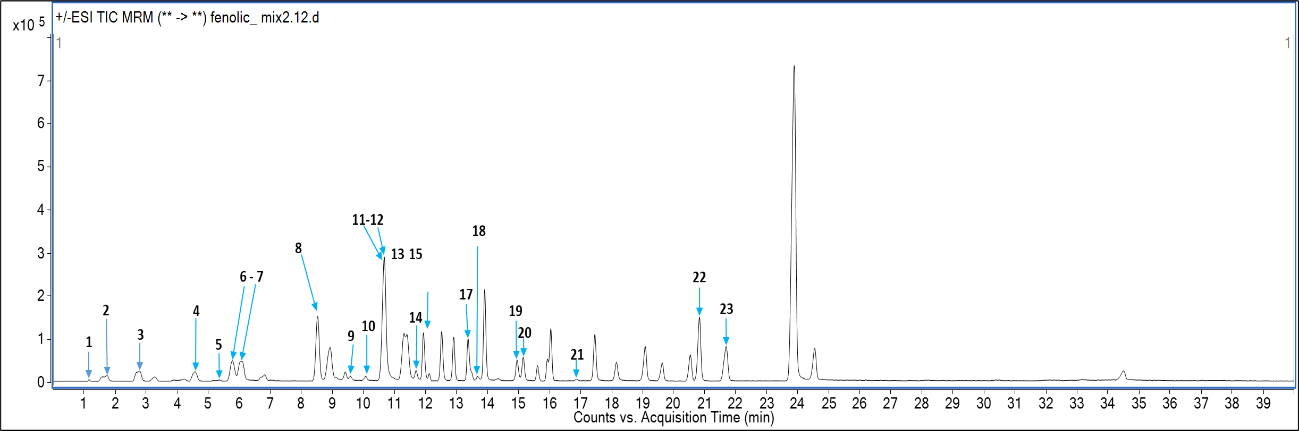


Figure 1. Standard chromatogram of phenolic compounds read in LC-MS/MS system (1-Shikimic acid, 2-Gallic acid, 3-Protocatechuic acid, 4-4-Hydroxybenzoic acid, 5-Chlorogenic acid, 6-Vanillic acid, 7-Caffeic acid, 8-*p*-coumaric acid, 9-Trans-ferrulic acid, 10-Sinapic acid, 11-Quercimerythrin, 12-Coumarin, 13-Scutellarin, 14-Hyperoside, 15-Rutin, 16-Isoquercetin, 17-Astragalin, 18-Fisetin, 19-Quercetin, 20-Naringenin, 21-Kaempferol, 22-Chrysin, 23-Flavone).

Figure 2. Chromatograms of phenolic compounds in asparagus spears according to regions

Figure 3. Spectrophotometric standard curves of DPPH IC50 in asparagus spears

Figure 4. Spectrophotometric standard curves of ABTS IC50 in asparagus spears


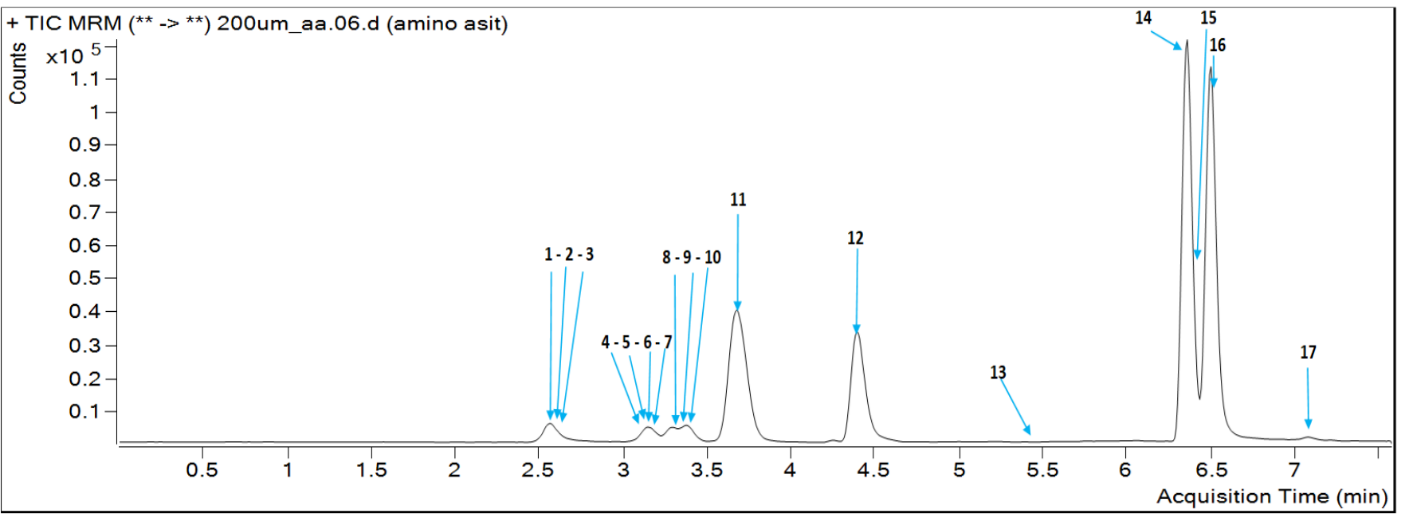


Figure 5. Standard chromatogram of amino acids read in the LC-MS/MS system (1- Lysine, 2- Histidine, 3- Arginine, 4- Cystine, 5- Glycine, 6- Serine, 7- Asparagine, 8- Threonine, 9- Aspartic acid, 10- Glutamic acid, 11- Proline, 12- Valine, 13- Methionine, 14- Tyrosine, 15- Isoleucine, 16- Leucine, 17- Phenylalanine).

Figure 6. Chromatograms of amino acids in asparagus shoots according to regions


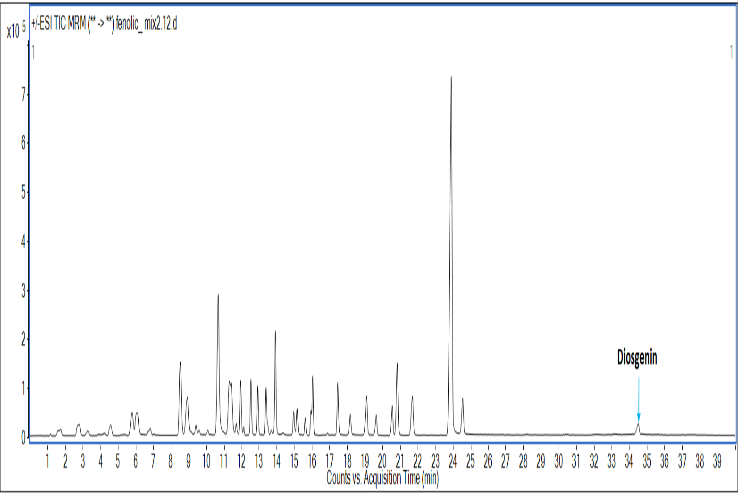


Figure 7. Diosgenin standard chromatogram in LC/MS-MS system and steroidal diosgenin standard curve used in saponin calculation.
